# Patient-level interventions to reduce alcohol-related harms in low- and middle-income countries: A systematic review and meta-summary

**DOI:** 10.1371/journal.pmed.1003961

**Published:** 2022-04-12

**Authors:** Catherine A. Staton, João Ricardo Nickenig Vissoci, Deena El-Gabri, Konyinsope Adewumi, Tessa Concepcion, Shannon A. Elliott, Daniel R. Evans, Sophie W. Galson, Charles T. Pate, Lindy M. Reynolds, Nadine A. Sanchez, Alexandra E. Sutton, Charlotte Yuan, Alena Pauley, Luciano Andrade, Megan Von Isenberg, Jinny J. Ye, Charles J. Gerardo

**Affiliations:** 1 Duke Division of Emergency Medicine, Department of Surgery, Duke University Medical Center, Duke University, Durham, North Carolina, United States of America; 2 Duke Global Health Institute, Duke University, Durham, North Carolina, United States of America; 3 Health Sciences Graduate Program, State University of Maringa, Maringa, Parana State, Brazil; 4 Nicholas School of the Environment, Duke University, Durham, North Carolina, United States of America; 5 Duke School of Medical Center Library Services & Archives, Duke University, Durham, North Carolina, United States of America; Massachusetts General Hospital, UNITED STATES

## Abstract

**Background:**

Disease and disability from alcohol use disproportionately impact people in low- and middle-income countries (LMICs). While varied interventions have been shown to reduce alcohol use in high-income countries, their efficacy in LMICs has not been assessed. This systematic review describes current published literature on patient-level alcohol interventions in LMICs and specifically describes clinical trials evaluating interventions to reduce alcohol use in LMICs.

**Methods and findings:**

In accordance with PRISMA, we performed a systematic review using an electronic search strategy from January 1, 1995 to December 1, 2020. Title, abstract, as well as full-text screening and extraction were performed in duplicate. A meta-summary was performed on randomized controlled trials (RCTs) that evaluated alcohol-related outcomes. We searched the following electronic databases: PubMed, EMBASE, Scopus, Web of Science, Cochrane, WHO Global Health Library, and PsycINFO. Articles that evaluated patient-level interventions targeting alcohol use and alcohol-related harm in LMICs were eligible for inclusion. No studies were excluded based on language.

After screening 5,036 articles, 117 articles fit our inclusion criteria, 75 of which were RCTs. Of these RCTs, 93% were performed in 13 middle-income countries, while 7% were from 2 low-income countries. These RCTs evaluated brief interventions (24, defined as any intervention ranging from advice to counseling, lasting less than 1 hour per session up to 4 sessions), psychotherapy or counseling (15, defined as an interaction with a counselor longer than a brief intervention or that included a psychotherapeutic component), health promotion and education (20, defined as an intervention encouraged individuals’ agency of taking care of their health), or biologic treatments (19, defined as interventions where the biological function of alcohol use disorder (AUD) as the main nexus of intervention) with 3 mixing categories of intervention types. Due to high heterogeneity of intervention types, outcome measures, and follow-up times, we did not conduct meta-analysis to compare and contrast studies, but created a meta-summary of all 75 RCT studies. The most commonly evaluated intervention with the most consistent positive effect was a brief intervention; similarly, motivational interviewing (MI) techniques were most commonly utilized among the diverse array of interventions evaluated.

**Conclusions:**

Our review demonstrated numerous patient-level interventions that have the potential to be effective in LMICs, but further research to standardize interventions, populations, and outcome measures is necessary to accurately assess their effectiveness. Brief interventions and MI techniques were the most commonly evaluated and had the most consistent positive effect on alcohol-related outcomes.

**Trial registration:**

Protocol Registry: PROSPERO CRD42017055549

## Abstract 2

Catherine Staton and co-workers report on evidence about interventions against harmful alcohol use in low- and middle-income countries.

## Author summary

**Why was this study done?:**

- Low- and middle-income countries (LMICs) report high rates of risky alcohol use behavior, a known risk factor for death and disability worldwide.
- In order to investigate the potential for a patient-level intervention to reduce alcohol-related harms in a low-income setting, we sought to identify interventions with adequate efficacy.

**What did the researchers do and find?:**

- We conducted a systematic review of studies from 1995 to 2020 in LMICs evaluating interventions to reduce alcohol use and alcohol-related harms.
- Of the 117 studies included for review, the majority were in middle-income countries and had varied intervention types, outcome measures, and follow-up time.
- The most commonly studied interventions with the most consistently positive results were brief interventions. Similarly, motivational interviewing (MI) techniques were the most commonly described intervention techniques.

**What do these findings mean?:**

- Future research on alcohol use and alcohol harm reduction in LMICs may benefit from consistency of methodologies, studying similar populations, interventions, and alcohol-related harm reduction outcome measures.
- Especially in LMICs, further research on comparative effectiveness or implementation strategies delineating optimal interventions and target populations is needed.

## Introduction

Alcohol use is an important cause of chronic disease and injury. It is one of the top 5 risk factors for death and disability in the world [1–3]. The detrimental effects of alcohol use contribute to 3.3 million deaths and 139 million disability-adjusted life years (DALYs) lost globally each year [4]. Alcohol use has also been associated with risky behaviors, including crime, aggressive driving, interpersonal violence, and self-inflicted injury [5]. Such behaviors not only have harmful effects on the individual but also on the greater population [6]. Compared to high-income countries, low- and middle-income countries (LMICs) report higher rates of risky drinking behaviors, such as binge drinking and episodic drinking, as well as an earlier onset of alcohol consumption [4].

The World Health Organization (WHO) has placed an emphasis on the development and implementation of both policy-level and patient-level interventions to reduce harmful alcohol use in LMICs. While policy-level interventions are a crucial, cost-effective manner of reducing alcohol-related harms, context-appropriate, and effective patient-level interventions are also greatly needed to form multipronged alcohol harm reduction strategies [4]. A broad array of patient-level alcohol harm reduction interventions, such as brief interventions (for this paper, defined as any intervention ranging from advice to counseling, lasting less than 1 hour per session [7] up to 4 sessions [8], psychosocial interventions, and pharmacological treatments) have been found to be effective in high-resource settings [9,10]. Yet, alcohol use disorders (AUDs), characterized by moderate to severe alcohol abuse and dependence, remain a low priority of LMIC health systems [11]. Barriers, such as funding constraints, lack of policy, and low public awareness, often prevent access to psychosocial and pharmacological treatments that target AUDs [11]. Especially in some settings where alcohol use is culturally ingrained, adopting an alcohol harm reduction strategy, as opposed to focusing on abstinence, is crucial given the limited alcohol policy, health system treatments, and social support [12]. As such, WHO and *The Lancet* have recently issued calls to action to reduce hazardous alcohol use [4,13], yet the full scope of the evidence-based patient-level interventions to reduce harmful alcohol use in LMICs is missing from the literature. While narrative reviews of global alcohol-related harms have been published, we have found no systematic review conducted focusing on alcohol interventions specifically applicable to or evaluated in LMICs [1,11].

In order to address this gap, this paper aims to (1) review and describe the current published literature on patient-level alcohol interventions in LMICs; and (2) conduct a meta-summary of studies evaluating interventions to reduce alcohol use and harms in LMICs.

## Methods

### Protocol and registration

This systematic review is reported in accordance with the Preferred Reporting Items for Systematic Reviews and Meta-Analyses (PRISMA) Statement [14] (see S1 Table) and is registered in the PROSPERO database (International Prospective Register of Systematic Reviews) under the number CRD42017055549.

### Eligibility criteria

Our primary criterion for article consideration was a patient-level alcohol or alcohol-related harm reduction intervention in a LMIC, as defined by our PICOS framework: LMIC **P**articipants, patient-level **I**nterventions, **C**ompared to a control group, alcohol harm reduction **O**utcomes, all **S**tudy designs but focused on randomized clinical trials if there are enough. To be included, articles had to (1) evaluate a patient-level alcohol-related intervention’s ability to reduce an (2) alcohol-related outcome in a (3) LMIC and be (4) peer-reviewed and published between January 1, 1995 and December 1, 2020. Study locations had to be classified as LMICs according to World Bank criteria at the time of the search [15]. The search strategy was inclusive of multiple study designs (randomized controlled trials [RCTs], prospective/retrospective cohort, quasi-experimental, or secondary data analyses with before and after intervention comparison) in case there was a dearth of literature from LMIC settings. Articles were excluded if they were abstracts only, literature or systematic reviews, meta-analyses, or commentaries. If 2 studies used the same data, then the most recent data were included in the review.

### Information sources

We searched electronic databases (PubMed, EMBASE, Scopus, Web of Science, Cochrane, WHO Global Health Library, and PsycINFO) for articles that evaluated patient-level interventions aimed at reducing an alcohol-related outcome in LMICs. No studies were excluded for language. Additionally, we manually searched references and performed a citation analysis of the included articles using Web of Science and Google Scholar. Any citation that met the inclusion criteria based on the title and abstract was added.

### Search

The initial search consisted of the MeSH terms “alcohol drinking,” “low or middle income country,” and “intervention.” Search strategy demonstrates the search strategy used in PubMed, Embase, PsycINFO, and WHO Global Health Library databases (S1 Fig).

### Study selection

Six pairs of reviewers from the specified individuals (KA, TC, SE, DE, SG, CP, LR, NS, AS, CY, and AP) independently reviewed the titles and abstracts, and any inconsistencies regarding inclusion were resolved by a third reviewer (DG or CS). Abstracts that did not provide enough information to determine eligibility were retrieved for full-text evaluation. Reviewers independently evaluated full-text articles and determined study eligibility. Disagreements were solved by consensus, and if disagreement persisted, a third reviewer’s opinion was sought. After inclusion, we assessed each study for the study design. We reported all study designs in order to summarize the type and quality of study designs in the literature. Based on the large number of RCTs identified, we chose to narrow further analysis to RCTs.

### Quality of studies

Since our systematic review included studies of different designs (RCTs, nonrandomized intervention, prospective/retrospective cohort, quasi-experimental, or secondary data/cross-sectional with before and after comparison), we opted to perform a data quality assessment according to study design using the following approaches. STrengthening the Reporting of OBservational studies in Epidemiology (STROBE) indicators were used for reporting observational studies. Two scales were used for nonrandomized studies: the A Cochrane Risk Of Bias Assessment Tool for Non-Randomized Studies (ACROBAT-NRS) [16] and Newcastle–Ottawa scale (NOS) [17]. Cochrane’s revised risk-of-bias tool was used for randomized studies [18]. Finally, the Effective Practice and Organisation of Care (EPOC) suggested risk of bias indicators for interrupted time series studies (EPOC) [19]. We assigned risk of bias (low, moderate, and high risk) as suggested by the Cochrane Handbook [20] by study design. Studies were classified as (a) low risk of bias if all domains had low risk; (b) some concerns if at least 1 domain raised some concerns for bias; and (c) high risk of at least 1 domain was at high risk.

### Data extraction

Five pairs of reviewers independently conducted the data extraction, and any disagreements were resolved by a third reviewer. General characteristics of the studies were recorded, such as year of publication, location where the study took place, inclusion and exclusion criteria, and participant characteristics. In addition, information on alcohol-related outcome measures, intervention type, and intervention impact or effectiveness measured as an effect size of outcome measures was extracted. The main outcome measures were Alcohol Use Disorders Identification Test (AUDIT) and Alcohol, Smoking and Substance Involvement Screening Test (ASSIST) scores, Rutgers Alcohol Problem Index (RAPI), number of drinking days, number of heavy drinking days, number of binge drinking days, drinks per drinking day, percent remaining abstinent from drinking alcohol, and percent relapsed back into drinking alcohol.

### Data analysis

Initial evaluation of the papers indicated that a meta-analytical approach would result in high heterogeneity due to high methodological variability (e.g., outcome measures, study designs, and sample characteristics). Therefore, we conducted a meta-synthesis for all the included manuscripts, which qualitatively aggregated findings by grouping relevant findings into categories that represent the study’s objectives (e.g., effectiveness of alcohol intervention). No manuscripts were excluded based on quality. The process involved summarizing main results of each included paper and performing a thematic analysis. Emerging themes on types of intervention and outcomes were presented. Interventions were grouped by similarity into 4 types: brief interventions, psychotherapy and counseling, health promotion and education, and biomedical treatments.

Using WHO and National Institute on Alcohol Abuse and Alcoholism (NIAAA) descriptions of brief interventions, we defined brief interventions as any intervention ranging from advice to counseling, lasting less than 1 hour per session [7] up to 4 sessions [8] independent of how the original study defined brief intervention. Interventions including a one-on-one interaction with a counselor that lasted longer than a brief intervention or that included a psychotherapeutic component were defined as psychotherapy and counseling. Motivational interviewing (MI) techniques could be included as either a brief intervention or psychotherapy and counseling, depending on how long and over how many sessions the intervention took place. A study was considered health promotion and education, independent of the study’s definition, if an intervention encouraged individuals’ agency of taking care of their health, such as risk reduction skills and health education [21]. Biomedical treatments were used as a taxonomy to group studies that had the biological function of AUD as the main nexus of intervention, including brain stimulation and medicines.

## Results

### Study selection and description

**Fig 1:**
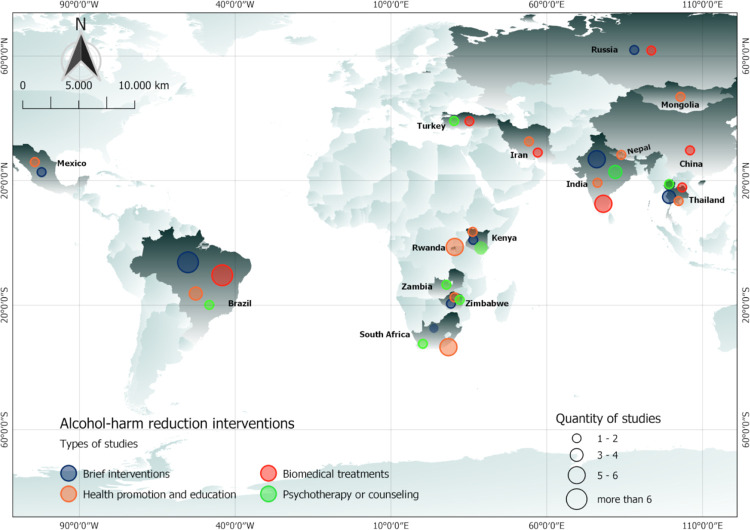
Study flow diagram.

In total, 5,036 abstracts were reviewed. From those, 500 articles were manually reviewed to identify 117 articles matching our inclusion and exclusion criteria (Fig 1). No studies were excluded based on language. Of these 117 studies, 75 were RCTs (Table 1) utilizing a vast array of interventions, which we categorized into 4 main categories of interventions, including brief interventions (24 studies) (Table 2), psychotherapy or counseling (15) (Table 3), health promotion and education (20) (Table 4), and biological treatments (19) (Table 5). One study by Shin and colleagues had one arm in biomedical treatments and another arm in brief intervention [22]. Two other studies had one arm in psychotherapy or counseling and another arm in health promotion and education [23,24]. These 75 studies were performed in 15 countries, representing 8 upper middle-income countries (60% of studies), 5 lower middle-income countries, and 2 low-income countries (7% of studies) (S2 Fig). The majority of the studies came from Brazil (28%) and India (20%). Alcohol-related outcomes found included alcohol quantity or frequency measure, intention to use alcohol, use/abstinence/remission proportion or frequency, alcohol-related scores, alcohol cravings or cravings per day, or alcohol use during pregnancy or before sex.

**Table 1:** Characteristics of all randomized controlled studies (75).

Authors	Country	Intervention type	Targeted population	Sample size	Risk of bias	Outcomes measured
Ahmadi and colleagues (2004) [24]	Iran	Biomedical treatments	Self-referred, alcohol-dependent males	116	High	Relapse
Ahmadi and colleagues (2019) [25]	Iran	Health promotion and education	Female drug users	100	High	Alcohol use before sexual intercourse
Aira and colleagues (2013) [26]	Mongolia	Health promotion and education	Power plant employees	200	Low	Drinking days Drinks per day
Altintoprak and colleagues (2008) [27]	Turkey	Biomedical treatments	AUD patient population	44	Low	Alcohol use Craving
Assanangkornchai and colleagues (2015) [28]	Thailand	Brief intervention	Primary care	236	Low	ASSIST Conversion to low risk
Babor and colleagues (1996) [29]	Australia, Kenya, Mexico, Norway, Wales, Russia, USA, and Zimbabwe	Brief intervention	Users at risk for dependence in hospital, emergency department, primary care, college, and health screening agency	1,559	High	Abstinence Frequency Intensity Harm (injury, legal problem, and unemployment) Complaint from others
Baldin and colleagues (2018) [30]	Brazil	Brief intervention	Nightclub users with drinking problems	465	Low	Binge drinking Lack of control
Baltieri and colleagues (2003) [31]	Brazil	Biomedical treatments	Alcohol-dependent males in outpatient treatment	75	Low	Abstinence
Baltieri and colleagues (2008) [32]	Brazil	Biomedical treatments	Alcohol-dependent males in outpatient treatment	155	High	Abstinence/relapse Weeks of heavy consumption
Barbosa Filho and colleagues (2019) [33]	Brazil	Health promotion and education	School-based adolescents	1,085	Low	Alcohol intake
Bedendo and colleagues (2019) [34]	Brazil	Brief intervention	College drinkers	4,460	Some concerns	AUDIT Alcohol-related consequences Drinks per drinking day
Bedendo and colleagues (2019) [35]	Brazil	Brief Intervention	College drinkers	5,476	Some concerns	AUDIT Alcohol-related consequences Drinking days Drinks per drinking days
Boggio and colleagues (2008) [36]	Brazil	Biomedical treatments	Alcohol-dependent users in rehabilitation program	13	Low	Alcohol Urge Questionnaire (craving level)
Bolton and colleagues (2014) [37]	Thailand	Health promotion and education	Survivors of imprisonment, torture, and related traumas	347	Low	Alcohol use
Burnhams and colleagues (2015) [38]	South Africa	Health promotion and education	Safety and security employees	325	Low	Binge drinking days Calling in sick or working with a hangover CAGE
Chaudhury and colleagues (2016) [39]	Rwanda	Health promotion and education	Families with caregiver HIV	293	Low	AUDIT
Chhabra and colleagues (2010) [40]	India	Health promotion and education	Teenage students	1,421	Low	Future intentions to use
Christoff and colleagues (2015) [41]	Brazil	Brief intervention	College students	815	Some concerns	ASSIST
Corrêa Filho and colleagues (2013) [42]	Brazil	Biomedical treatments	Alcohol-dependent males in outpatient treatment	102	Low	Drinks per day Abstinence Heavy drinking days
Cubbins and colleagues (2012) [43]	Zimbabwe	Health promotion and education	Rural communities	5,543	High	Abstinence Drinks per drinking day Drinking days Drunk days
da Silva and colleagues (2013) [44]	Brazil	Biomedical treatments	Alcohol-dependent users in outpatient treatment	13	Low	Relapse OCDS Alcohol Urge Questionnaire
Daengthoen and colleagues (2014) [45]	Thailand	Psychotherapy or counseling	Alcohol-dependent users in inpatient treatment	100	Low	Craving days Abstinent days Drinking days
De Sousa and colleagues (2004) [46]	India	Biomedical treatments	Private hospital adult psychiatric patients	100	High	Abstinence days Days until relapse Drinks per drinking day Craving
De Sousa and colleagues (2005) [47]	India	Biomedical treatments	Private hospital adult psychiatric patients	100	High	Abstinence days Days until relapse Drinks per drinking day Craving
De Sousa and colleagues (2008) [48]	India	Biomedical treatments	Private hospital adolescent psychiatric patients	100	High	Abstinence days Days until relapse Drinks per drinking day Craving
De Sousa and colleagues (2008) [49]	India	Biomedical treatments	Private hospital adult psychiatric patients	100	High	Abstinence days Days until relapse Drinks per drinking day Craving
De Sousa and colleagues (2014) [50]	India	Biomedical treatments	Private hospital adult psychiatric patients	100	High	Abstinence days Days until relapse Drinks per drinking day Craving
Furieri and colleagues (2007) [51]	Brazil	Biomedical treatments	Alcohol-dependent users referred for alcohol treatment	60	Low	Drinks per day Drinks per drinking day Heavy drinking days Percent abstinent OCDS
Gupta and colleagues (2017) [52]	India	Biomedical treatments	Alcohol-dependent users in outpatient treatment	122	Some concerns	Heavy drinking days Abstinent days Days to first relapse Relapse Abstinence OCDS
Hartmann and colleagues (2020) [53]	India	Psychotherapy or counseling	Couples	60 couples	Some concerns	Alcohol Breathalyzer Abstinence
Jirapramukpitak and colleagues (2020) [54]	Thailand	Health promotion and education	Alcohol-dependent users	161	Some concerns	Abstinence
Jordans and colleagues (2019) [55]	Nepal	Health promotion and education	Mental health patients at the primary care setting	162	Low	AUDIT
Kalichman and colleagues (2008) [56]	South Africa	Health promotion and education	Users at informal drinking establishment	353	Low	Alcohol outcome expectancy (“I am a better sex partner after I have been drinking” and “When I’m drinking, I do things I wouldn’t usually do”)
Kalichman and colleagues (2007) [57]	South Africa	Health promotion and education	Sexually transmitted infections clinic	143	Low	
Kamal and colleagues (2020) [58]	India	Brief Intervention	College students with hazardous use	130	Low	AUDIT
Klauss and colleagues (2014) [59]	Brazil	Biomedical treatments	Alcohol-dependent users	33	Low	Relapse OCDS
Klauss and colleagues (2018) [60]	Brazil	Biomedical treatments	Alcohol-dependent users	45	Low	Relapse OCDS
L’Engle and colleagues (2014) [61]	Kenya	Psychotherapy or counseling	Female sex workers	818	High	Drinks per week Binge drinking
Likhitsathian and colleagues (2013) [62]	Thailand	Biomedical treatments	Inpatient treatment for AUDs	106	Low	Heavy drinking days Drinks per day Drinks per drinking day Cravings (visual analog) Heavy drinking relapses
Madhombiro and colleagues (2020) [63]	Zimbabwe	Psychotherapy or counseling	HIV clinic	234	Low	AUDIT
Marques and colleagues (2001) [64]	Brazil	Psychotherapy or counseling	Alcohol-dependent users	155	High	Drinking days Heavy drinking days Problem drinking days Drinks per week Alcohol Dependence Data Questionnaire
Marsiglia and colleagues (2015) [65]	Mexico	Health promotion and education	Middle school students	431	Some concerns	Drinks per month Drinking days
Mendez-Ruiz and colleagues (2020) [66]	Mexico	Health promotion and education	Sexually active female college students	132	Some concerns	AUDIT
Mertens and colleagues (2014) [67]	South Africa	Brief intervention	Young adults from primary care clinic	403	Low	ASSIST Heavy drinking
Moraes and colleagues (2010) [23]	Brazil	Psychotherapy or counseling and Health promotion and education	Alcohol-dependent users in outpatient treatment	120	High	Abstinence Drinking days Addiction Severity Index
Murray and colleagues (2020) [68]	Zambia	Psychotherapy or counseling	Couples with male hazardous use and intimate partner violence	248 couples	Low	AUDIT
Nadkarni and colleagues (2015) [69]	India	Brief intervention	Males presenting to primary care	53	Low	AUDIT
Nadkarni and colleagues (2017) [70]	India	Brief intervention	Harmful drinking in males in primary care	377	Low	AUDIT Abstinence Amount of consumption Heavy drinking days
Nadkarni and colleagues (2017) [71]	India	Brief Intervention	Harmful drinking in males in primary care	377	Low	AUDIT Abstinence Amount of consumption
Nadkarni and colleagues (2019) [72]	India	Brief Intervention	Alcohol-dependent males	135	Low	Remission (AUDIT <8) Mean daily alcohol consumption % abstinent % heavy drinking days Uptake of detoxification services SIP
Nattala and colleagues (2010) [73]	India	Psychotherapy or counseling	Inpatient	90	High	Abstinence Amount of consumption Drinking days
Ng and colleagues (2020) [74]	India	Psychotherapy or counseling	Alcohol-dependent users	60	Some concerns	Craving Drinking days Drinks per drinking days Relapse
Noknoy and colleagues (2010) [75]	Thailand	Brief intervention	Harmful users in primary care	117	Low	Drinks per drinking day Hazardous drinking Drinks per week Binge drinking
Pal and colleagues (2007) [76]	India	Brief intervention	Male harmful users	90	Some concerns	Drinking days Addiction Severity Index
Papas and colleagues (2011) [77]	Kenya	Psychotherapy or counseling	HIV clinic	75	Low	Drinking days Drinks per drinking day Abstinence
Papas and colleagues (2020) [78]	Kenya	Psychotherapy or counseling and Health promotion and education	HIV clinic	614	Low	% drinking days Drinks per drinking day
Peltzer and colleagues (2013) [79]	South Africa	Brief intervention	TB patients	853	Low	AUDIT Heavy episodic drinking
Pengpid and colleagues (2013a) [80]	South Africa	Brief intervention	Hazardous or harmful users	392	Some concerns	AUDIT Heavy episodic drinking
Pengpid and colleagues (2013b) [81]	South Africa	Brief intervention	University students	152	Some concerns	
Pengpid and colleagues (2015) [82]	Thailand	Brief intervention	Outpatient clinic	620	Low	Drinks per week ASSIST
Rendall-Mkosi and colleagues (2013) [83]	South Africa	Psychotherapy and counseling	Pregnant women	165	Some concerns	Risky drinking AUDIT
Rotheram-Borus and colleagues (2015) [84]	South Africa	Health promotion and education	Pregnant women	904	Some concerns	Drinking days Drinks per drinking day Heavy drinking days
Sanchez and colleagues (2017) [85]	Brazil	Health promotion and education	Early adolescents (seventh and eighth grades)	5,028	Low	Binge drinking episodes
Sanchez and colleagues (2018) [86]	Brazil	Health promotion and education	Early adolescents (seventh and eighth grades)	5,028	Low	Binge drinking episodes
Satyanarayana and colleagues (2016) [87]	India	Psychotherapy and counseling	Alcohol-dependent males in inpatient treatment	177	Low	SADQ
Segatto and colleagues (2011) [88]	Brazil	Brief intervention	Young adults presenting to emergency department	175	Low	Drinking days Light, moderate, or heavy drinking days RAPI ACRQ
Shin and colleagues (2013) [22]	Russia	Biomedical treatments and Brief intervention	Adults hospitalized for TB	196	Some concerns	Abstinent days Heavy drinking days
Signor and colleagues (2013) [89]	Brazil	Brief intervention	Callers to counseling hotline	637	High	% abstinent
Simao and colleagues (2008) [90]	Brazil	Brief intervention	University students	266	Some concerns	RAPI AUDIT Brief Drinker Profile Alcohol Dependence Scale
Soares and Vargas (2019) [91]	Brazil	Psychotherapy and counseling	Harmful or hazardous users	180	High	AUDIT
Sorsdahl and colleagues (2015) [92]	South Africa	Psychotherapy and counseling	Emergency department	335	Low	ASSIST
Ward and colleagues (2015) [93]	South Africa	Brief intervention	Young adults in primary care	363	Low	ASSIST
Wechsberg and colleagues (2019) [94]	South Africa	Brief intervention	Black African women going through HIV prevention	641	Low	Frequency of heavy drinking episode
Witte and colleagues (2011) [95]	Mongolia	Health promotion and education	Female sex workers	166	Low	AUDIT
Zhao and colleagues (2020) [96]	China	Biomedical treatments	Alcohol-dependent males with withdrawal symptoms	62	Some concerns	Craving (PACS)

ACRQ, Alcohol Consumption Risk Questionnaire; ASSIST, Alcohol, Smoking and Substance Involvement Screening Test; AUD, alcohol use disorder; AUDIT, Alcohol Use Disorders Identification Test; BrAC, breath alcohol content; CAGE, Cut, Annoyed, Guilty, and Eye questionnaire; OCDS, Obsessive Compulsive Drinking Scale; PACS, Penn Alcohol Craving Scale; SADQ, Severity of Alcohol Dependence Questionnaire; SIP, Short Inventory of Problems; TB, tuberculosis.

**Table 2:** Meta-synthesis of studies assessing patient-level interventions to reduce alcohol harms in LMICs: brief intervention RCTs.

Intervention description	Follow-up time	Outcomes	Summary of findings
WHO-based brief interventions (which uses MI techniques) (6)	3, 6, 9, and 12 months	Harmful alcohol use score (AUDIT) Alcohol misuses (ASSIST) Heavy drinking % of remission (ASSIST) % abstinent % daily or almost daily drinking % above recommended weekly limit % hazardous average daily consumption	At 3 and 6 months, Assanangkornchai and colleagues found similar significant reductions in the frequency of alcohol use and other substances in both the intervention and control groups at the primary care setting [28]. Penpgid and colleagues did not find evidence of efficacy of a mixed alcohol and tobacco brief intervention compared to an alcohol-only or tobacco-only session on past week alcohol use and Alcohol ASSIST score. All 3 arms did have a significant reduction in their alcohol consumption compared to baseline [82]. Babor and colleagues found at 9 months that males who received brief or simple advice reported a 17% lower average daily alcohol consumption compared to the control group, with a reduction in the intensity of drinking at about 10%. Females reduced their consumption in both groups without between-group differences [29]. Pengpid and colleagues found that at 12 months postintervention, university students had a significant reduction in AUDIT score compared to control [81]. As for outpatients, they found no significant differences in the reduction in relation to control [80]. Peltzer and colleagues evaluated the impact of a brief intervention versus a health leaflet for TB clinic patients and did not find evidence of efficacy between control and intervention at 6 months (79).
Face to face and computer based (1)	3 months	ASSIST	At 3 months, a face-to-face and computer-based MI both reduced ASSIST scores compared to the control group with computer-based intervention with the greater reduction [41].
Nurse, nurse practitioner, or lay counselor delivered (5)	6 weeks 3, 6, and 12 months	ASSIST % heavy drinking % at-risk use Binge drinking days Drinking days Drinks per day Heavy drinking	Mertens and colleagues found those who received a nurse practitioner–delivered brief intervention reduced patients’ alcohol ASSIST scores at 3 months by 38% versus 21% in the control arm [67]. At 3 months, Noknoy and colleagues found a significant difference in number of binge drinking days between intervention (0.29) and control group (1.36), but at 6 weeks and 6 months, there was no significant difference. At 6 weeks, 3 months, and 6 months, there were significant differences in the average drinks per drinking day between intervention (3.00, 2.73, and 2.26) and the control group (4.85, 5.06, and 4.02), but no difference in the number of drinking days between baseline and follow-up [75]. Nadkarni and colleagues found 36% remission (AUDIT 12–19) of alcohol use in the intervention group compared to the 26% of the control group. At 3 months follow-up, abstinence was significantly higher in the intervention (42%) compared to control (18%) groups. No effect on mean daily alcohol consumption or percent days of heavy drinking differences was found [70]. Results at 12 months showed maintained and enhanced effects on alcohol-related outcomes [71]. A pilot study found that for men with AUDIT>20, the CAP intervention arm had nonsignificant favorable outcomes for remission, proportion of nondrinkers, and ethanol consumption at 3- and 12-month follow-up as compared to enhanced usual care [72].
MI (6)	1, 3, and 6 months	Alcohol Consumption Questionnaire RAPI score ACRQ APRA Alcohol abstinence ASSIST Drinking days ASI	Segatto and colleagues found significant reduction in alcohol-related problems and alcohol use in the brief intervention and alcohol educational brochure groups but no significant differences between the groups for days of use and amount of use, RAPI, ACRQ and APRA scores, at 3 months follow-up [88]. Signor and colleagues found a significant difference between groups in the reduction of participants consuming alcohol at 6 months follow-up (70% of individuals in the helpline-based brief intervention group and 41% in the control/minimal intervention group) [89]. Ward and colleagues found that those who received a brief MI at the primary care setting and resource list were more likely to reduce alcohol misuse than control at 3 months [93]. Pal and colleagues found men who received a brief intervention had a decreased average amount of alcohol use in prior 30 days (24.7 to 10.1 versus 26.1 to 19.1) and decreased Addiction Severity Index (0.36 to 0.18 versus 0.42 to 0.33) at 3 months compared to those who received simple advice only [76]. A significant reduction in AUDIT scores at 3 months follow-up was observed by Kamal and colleagues for an on-campus, nurse-delivered brief alcohol screening, and intervention as compared to general advice. The intervention group also had a significant shift of participants from high- to low-risk AUDIT zone as compared to the control group [58]. Shin found no differences in the proportion of abstinent days between intervention and control in a TB clinic [22].
BASICS, MI, and harm reduction (1)	12 and 24 months	# drinks per day RAPI score Harmful alcohol use score (AUDIT)	Simao and colleagues found that college students receiving a brief alcohol screening and intervention had a decrease in the quantity of alcohol use per occasion (4.5 drinks/occasion to 3.7) compared to control (5.1 drinks/occasion to 5.0) at 24 months. There was also significant reduction in AUDIT and RAPI scores between intervention (9.6 to 7.3; 7.0 to 4.3) and control (9.6 to 8.6; 7.6 to 3.9, respectively) [90].
PNF (3)	1, 3, and 6 months	AUDIT/AUDIT-c Alcohol consequences # of drinks Binge drinking	The intervention group showed a reduction in the number of drinks in a typical drinking day at all follow-up times (OR ranging from 0.71 to 0.68) compared to control. A significant increase in alcohol consequences was observed in the intervention group at 3 months compared to control. The intervention effects were higher for participants with higher motivation for receiving the intervention groups [34]. No differences in binge drinking between control and intervention were observed by Baldin and colleagues [30]. Bedendo and colleagues found in this we-based study of college students a significant reduction in AUDIT scores among NFO and CFO study arms at 1 and 3 months follow-up, respectively, as compared to the PNF arm. Alcohol consequences were lower in NFO at 1 month follow-up and in drinking frequency at 3 months follow-up compared to PNF [35].
Women-focused social cognitive oriented behavioral intervention (1)	6 and 12 months	Heavy drinking episodes # binge drinking days	Intervention arm showed significantly less frequent heavy drinking behavior (−13.5 in % points) and heavy drinking days (9.9 [SD 8.4] average drinks for control versus 7.4 [SD 7.8] for intervention) at 6 months, but no changes at 12 months. There was no difference in the average number of drinks per drinking days at both follow-up times [94].

ACRQ, Alcohol Consumption Risk Questionnaire; APRA, Alcohol Perception of Risk Assessment; ASI, Alcohol Severity Index; ASSIST, Alcohol, Smoking and Substance Involvement Screening Test; AUDIT, Alcohol Use Disorders Identification Test; BASICS, Brief Alcohol Screening and Intervention of College Students; CAP, Counseling for Alcohol Problems; CFO, consequences feedback only; LMIC, low- and middle-income country; MI, motivational intervention; NFO, normative feedback only; OR, odds ratio; PNF, personalized normative feedback; RAPI, Rutgers Alcohol Problem Index; RCT, randomized controlled trial; TB, tuberculosis; WHO, World Health Organization.

**Table 3:** Meta-summary of studies assessing patient-level interventions to reduce alcohol harms in LMICs: psychotherapy or counseling RCTs.

Intervention description		Follow-up times	Outcomes	Summary of findings
CBT (6)	Individual versus group CBT	15 months	# binge drinking days # drinking per drinking days % harmful drinking	Marques and colleagues found that at 15 months, both group and individual interventions had reduction in the mean number of drinking days (group 51 to 29 and 47 to 30), number of heavy drinking days (40 to 20 and 29 to 11), number of problem drinking days (21 to 7 and 12 to 4), mean weekly consumption (43 to 19 and 30 to 12), GGT (109 to 43 and 87 to 34), and SADD (17 to 11 and 17 to 11). There was no difference between the groups [64].
	CBT	1, 2, and 3 months	SADQ scores # drinking days	Satyanarayana and colleagues found that both usual care and CBT for inpatient alcohol-dependent males who screened positive for intimate partner violence reduced SADQ scores over 3 months (ICBI 28.9 to 18.9, 27.3 to 19.7) with no significant between-group differences [87]. Papas and colleagues found that compared to usual care, CBT for HIV-infected outpatients who reported hazardous or binge drinking showed a reduction in mean difference percent drinking days (24.9) and drinks per drinking days (2.88) at 30 days follow-up [77].
	CETA, a CBT-based treatment model targeting mental and behavioral comorbidities	12 months	AUDIT	At 12 months follow-up, Murray and colleagues found a significantly greater reduction in the mean AUDIT score of the CETA intervention arm (14.9 to 5.7) compared to treatment as usual (14.6 to 10.0) in couples with intimate partner violence [68].
	Group CBT versus healthy lifestyle education	9 months	% drinking days Drinks per drinking day	Papas and colleagues found that compared to healthy lifestyle education, the group CBT intervention arm had significantly lower % drinking days (10.26 versus 7.58) and drinks per drinking day (1.69 versus 1.15) overall [78].
	CBT with CM	1 month	BrAC	Hartmann and colleagues found that compared to usual care, a significantly greater proportion of individuals receiving CBT with incentive-based CM tested negative for alcohol consumption (0.96 versus 0.76) at 1 month follow-up; incentives-only arm had a similar reduction in alcohol consumption to the CBT with incentive-based CM [53].
Combined methods (5)	Phramongkutklao model, an inpatient rehabilitation program using Buddhism, CBT, health education, family education, and relaxation therapy	1, 3, and 6 months	Abstinent days Alcohol consumption Craving days	Daengthoen and colleagues found an intensive inpatient rehabilitation model (PMK) found a significant difference in the mean difference of alcohol consumption (mean difference −9.4 baseline, −23.0 1 month, −3.3 3 months, and −4.4 6 months) and mean drink cravings (4.3 versus 3.3) at 1, 3, and 6 months [45].
	Family inclusive relapse prevention	6 months	% of abstainers days	Nattala and colleagues found a significantly higher percentage of dyadic relapse prevention patients were abstinent throughout the 6-month follow-up period (57%) compared to individual relapse prevention (27%) and treatment as usual (30%) [73].
	MI and PST	3 months	Harmful alcohol use score (AUDIT)	Sorsdahl and colleagues found for emergency department patients, there was a significant reduction in substance use determined by ASSIST at 3 months for those who received a MI–PST intervention (18.71 to 9.89) compared to the MI (19.96 to 12.28) and control (19.3 to 11.91). There was no significant difference between the MI and control group [92].
	Combined MI and CBT nurse delivered individual counseling	6 months	AUDIT	At 6-month follow-up, Madhombiro and colleagues found a significantly greater change in AUDIT scores in the intervention arm (14.89 to 8.75) as compared to enhanced usual care (14.74 to 11.61) [63].
	BMS intervention (1), Multidimensional holistic group intervention combining health education and relapse prevention with acupuncture, breathing, and meditation-based exercises	1, 2, and 3 months	PACS Drinking days Drinks per drinking day Relapse	Ng and colleagues found significantly less alcohol cravings, drinking days, drinks per drinking day, and rates of relapse in the BMS intervention group as compared to treatment as usual at 3-month follow-up [74].
MI (4)	MI based counseling sessions, WHO Brief Intervention for Alcohol Use	6 and 12 months	% of abstinent days # binge drinking days	At 6 months, there were significant reductions in alcohol use over the prior 30 days for the intervention group with 53.8% reporting never drinking over the prior 30 days compared to 26.2% of the control group. Significant reduction in binge drinking with 73.7% of the intervention group compared to 33.2% of the control group reporting never binge drinking in the prior 30 days [61]. At 12 months, 66.3% of the intervention group reported never drinking over the prior 30 days compared to 39.4% of the control group. Similarly, 78.9% of the intervention group reported never binge drinking compared to 47.6% of the control group [61].
	Group-based MI	3 and 12 months	AEP % harmful alcohol use (risky drinking)	Rendall-Mkosi and colleagues found that compared to the control, a 5-session intervention reduced the proportion of women at risk for AEP (51% intervention and 28% control) at 3 and 12 months. There were declines for both groups in the proportion of women who met criteria for risky drinking at 3 and 12 months (intervention 14.75% versus 10.94%), but the difference between the 2 groups was not significant [83].
	Relapse prevention and MI with or without HVs for outpatients	3 months	% abstinence Consumption days	Moraes found that after intensive outpatient intervention, of those with subsequent HVs 51.8% were abstinent compared to 43.1% being abstinent among those with no HV controls at 3 months follow-up [23].
	NIH/NIAA-based brief counseling	6 months	Abstinent days Heavy drinking days	Shin found that for hospitalized TB patients with AUDs who were given a brief counseling intervention with or without naltrexone, there was no change in mean number of abstinent days in the prior 30 days nor number of heavy drinking days [22].

AEP, alcohol-exposed pregnancy; AUD, alcohol use disorder; AUDIT, Alcohol Use Disorders Identification Test; BMS, body–mind–spirit; BrAC, breath alcohol concentration; CBT, cognitive behavioral therapy; CETA, Common Elements Treatment Approach; CM, contingency management; GGT, Gamma-Glutamyl Transferase; HV, home visit; ICBI, integrated cognitive-behavioral intervention; LMIC, low- and middle-income country; MI, motivational interviewing; NIAA, National Institute on Alcohol Abuse and Alcoholism; NIH, National Institutes of Health; PACS, Penn Alcohol Craving Scale; PMK, Phramongkutklao; PST, problem solving therapy; RCT, randomized controlled trial; SADD, Short Alcohol Dependence Data Questionnaire; SADQ, Severity of Alcohol Dependence Questionnaire; TB, tuberculosis; WHO, World Health Organization.

**Table 4:** Meta-summary of studies assessing patient-level interventions to reduce alcohol harms in LMICs: health promotion and education RCTs.

Intervention description		Follow-up times	Outcomes	Summary of findings
Workplace health promotion programs (2)	Team awareness, social cognitive, and MI theory health promotion	3 months	Binge drinking # drinking days # drinking per days Attitude toward alcohol	Burnhams and colleagues found that a team awareness intervention reduces the mean binge drinking days from 2.1 to 1.4 days compared to an increase from 1.6 to 2.1 days in the control group [38]. Aira and colleagues found at a 3-month follow-up, a health promotion intervention for factory workers had a reduction in alcohol drinks per day for men (b −0.19) and women (b −0.28) and attitudes toward drinking (b 3.06), but not for days of alcohol consumption (b −0.13) [26].
Community based (5)	Community-based intervention for risk reduction of HIV-related behaviors	12 and 24 months	Current alcohol use Frequency of alcohol use Quantity of drinks consumed	Cubbins and colleagues studied a community-based intervention and found declines in alcohol use and abuse over the study period in relatively equal levels [43].
	HV HV with CM HV for pregnant women	12, 13, and 16 weeks 18 and 36 months	Abstinence Drinking days Alcohol use during pregnancy Frequency of use # of drinks per drinking day Frequency of 3 or more drinks per day AUDIT	Moraes and colleagues evaluated the cost-effectiveness of an outpatient conventional (CT) alcohol rehabilitation treatment to conventional treatment with HV. Authors found both groups had a large proportion of the patients were abstinent at follow-up CT (3.4% to 43.1%) and HV (1.6% to 58.11%), but the overall difference of 44% more abstinent patients was not significantly different [23]. Jirapramukpitak and colleagues found that home-based CM did not improve continuous abstinence over the 12-week intervention period. The higher-magnitude CM intervention arm did have a significantly higher abstinence rate in the postintervention follow-up period [54]. Rotheram-Borus and colleagues studied a HV for prenatal and postnatal visits for pregnant women up to 36 months postdelivery and did not find a direct association between intervention and alcohol use [84]. Bolton and colleagues found no differences in AUDIT scores between a control and intervention in Burmese refugees in Thailand [37].
School based (6)	STEP for HIV/AIDS and alcohol use	10 weeks	Intention to use	Chhabra and colleagues found no differences in intention to use alcohol after implementation of a STEP program compared to control at 10-week outcome assessment [40].
	School-based curriculum using communication competence theory to develop use resistance strategies	8 months	Drinks per day Drinking days	Marsiglia and colleagues found that after an implementation of a school-based curriculum, both intervention and control groups had an increase in the amount of use and frequency of use, yet the intervention group had significantly less increase in amount and frequency of use [65].
	Socioecological theory and sociocognitive theory–based healthy lifestyle education and environmental changes	4 months	% participants reporting alcohol intake	Barbosa Filho and colleagues found that no differences between control and intervention groups were observed in the proportion of adolescents reporting not taking alcohol in the last month [33].
	Nurse-delivered Health, Education, Prevention and Self-Care (SEPA) based on Social Cognitive Model of Behavior Change	1 month	AUDIT	Among sexually active university-recruited women, Mendez-Ruiz and colleagues found decreased alcohol use in the intervention group compared to the control group [66].
	Life skills development curriculum for schools based on a comprehensive social influence program	9 and 21 months	% of first use of alcohol # of binge drinking days % of alcohol use	No differences were observed at 9 months between intervention and control for alcohol use and binge drinking. At 9 months, participants in the intervention group showed a higher chance of using alcohol for the first time (RR 1.30, CI 95% 1.13;1.49) [85]. At 21 months, participants in the intervention group reported higher risk of initiating alcohol use (OR 1.13, CI 95% 1.01;1,27) and higher chance of using alcohol in the past year (OR 1.30, CI 95% 1.02;1.65). No effects were observed for binge drinking in the past year or alcohol use and binge drinking in the past month [86].
Clinic based (7)	Family Strengthening Intervention for HIV-affected families	3 months	Caregiver AUDIT	Chaudhury and colleagues found compared to treatment as usual, a family-based intervention to reduce alcohol use and violence within HIV-affected families in Rwanda had had significant reductions in alcohol use compared to control (−0.56) at 3-month follow-up [39].
	HIV–alcohol risk reduction intervention	1, 3, and 6 months	Alcohol use in sexual context Anticipated outcome of alcohol use	Kalichman and colleagues found that a behavioral risk reduction counseling intervention for sexually transmitted infection clinic patients had a reduction in alcohol use and expectancies that alcohol enhances sexual experiences at 3-month follow-up [57]. Kalichman and colleagues found that compared to a 1-hour HIV–alcohol education group, the 3-hour brief behavioral HIV–alcohol risk reduction intervention reduced alcohol use before sex at 3 and 6 months [56]. Ahmadi and colleagues found that, compared to treatment as usual, an HIV-focused peer education training program had significant reductions in both alcohol use prior to sexual intercourse and number of sex acts while intoxicated among female drug users at 1 and 3 months follow-up [25].
	Group CBT versus healthy lifestyle education	9 months	% drinking days Drinks per drinking day	Papas and colleagues found that compared to healthy lifestyle education, the group CBT intervention arm had significantly lower % drinking days (10.26 versus 7.58) and drinks per drinking day (1.69 versus 1.15) overall [78].
	HIV SR reduction arm and MI+risk reduction	3 and 6 months	AUDIT	Witte and colleagues studied the efficacy of a relationship-based SR reduction intervention, SR reduction intervention with MI compared to a wellness control to reduce harmful alcohol use among female sex workers. All groups were effective in reducing the AUDIT score from baseline to 6 months (wellness promotion −30.98 to 18.30, risk reduction −28.42 to 18.12, and risk reduction and MI −32.64 to 21.72), but there was no significant difference between groups [95].
	Multifaceted district level mental healthcare plan + brief intervention	12 months	AUDIT	Jordans and colleagues found no statistical significant difference between control and intervention for the reduction in AUDIT scores from baseline and follow-up (B = 12.16; CI 95% −6.10; 1.79) [55].

AUDIT, Alcohol Use Disorders Identification Test; CM, contingency management; HV, home visit; LMIC, low- and middle-income country; MI, motivational interviewing; OR, odds ratio; RCT, randomized controlled trial; RR, risk ratio; SEPA, Health, Education, Prevention and Self-Care; SR, sexual risk; STEP, School-based Teenage Education Program.

**Table 5:** Meta-summary of studies assessing patient-level interventions to reduce alcohol harm in LMICs: biological treatment RCTs.

Intervention description		Follow-up times	Outcomes	Summary of findings
Medication (15)	Naltrexone (3)	1, 2, 3 and months	% abstinent % relapse # abstinent days # heavy drinking days	There was no significant difference in the percentage of abstinence, number of heavy drinking days, or number of abstinent days when comparing naltrexone and the placebo [22,24,32]. Naltrexone significantly decreased the relapse percent when compared with the placebo, 74.14% relapse in control, versus 55.17% relapse in the intervention. [24], although another study reported no significant change in percent abstinent at fourth week or eighth week (intervention: week 4: 53.1% week 8: 40.8% Control: week 4: 42.6% week 8: 31.5%) [32].
	Acamprosate with participation in AA optional (1)	1, 2, 3, 4, 6, 8, 12, 16, 20, and 24 weeks	% abstinent Abstinent days	There was a significant difference in abstinence between the trial group (acamprosate) (42.5%) and the control group (20%) [31]. Abstinent days were significantly greater in patients who received acamprosate and did not participate in AA than in patients who received placebo and did not participate in AA. Abstinent days were not significantly greater in the subgroup who received acamprosate and participated in AA than in the subgroup who received placebo and participated in AA [31].
	Ondansetron (1)	3 months	% abstinent # drinks per day	There was no significant difference between the trial group (ondansetron) and the control group (placebo) for the main outcome, percentage of study participants abstinent (trial: 88.6%, placebo: 76.1%). There was also no significant difference between mean number of drinks per day (trial: 0.66, placebo: 1.09) [42].
	Baclofen + brief intervention (1)	3 months	# abstinent days	Baclofen and brief intervention (FRAMES) significantly increased the number of abstinent days (65.1) when compared to the benfotiamine (nutritional supplement/control) group and brief intervention (FRAMES) (39.66) [52].
	Gabapentin (1)	1 month	Drinks per days Drinks per drinking day % heavy drinking % abstinent OCDS	The gabapentin group had a significantly decreased number of drinks per day, weekly drinks, alcohol consumption during 4 weeks of treatment, and mean percentage of heavy drinking days, and a significantly higher mean percentage of days of abstinence. No differences in drinks per drinking day or OCDS scores between groups [51].
	Topiramate (1, 1 repeated sample)	1, 2, and 3 months	% abstinent # drinks per day # drinks per drinking day % drinking days # heavy drinking days # abstinent days	Topiramate caused a significant increase in the percentage of abstinence at 4 weeks compared to the control group, 42.6% in the placebo and 67.3% in intervention [32]. There was no statistical difference between topiramate and the placebo at week 4, week 8, and week 12 for percent heavy drinking days (intervention: 0.7, 4.9, 2.3; control: 5.0, 5.7, 5.3), percent of drinking days (intervention: 6.6, 7.5, 5.5; control: 11.9, 11.3, 6.4), number of drinks per drinking day (intervention: 1.1, 2.9, 1.2; control: 1.7, 2.2, 4.2), and number of drinks per day (intervention: 0.2, 0.7 0.7; control: 0.7, 0.7, 0.9) [62].
	Disulfiram (5)	9 and 12 months	# of days of abstinence # days until relapse # drinks per week # drinks per occasion OCDS	The groups receiving disulfiram showed higher frequency of days of abstinence, higher days to first relapse, less craving and less relapse events than topiramate in alcohol-dependent men [49]. A similar pattern of results were observed when comparing disulfiram to naltrexone, but had higher cravings. No differences were observed in the amount of days to the first alcohol used [46,48,50]. Compared to acamprosate, the group receiving disulfiram showed higher abstinent days, fewer relapse events, and a higher number of days until first alcohol use and to first relapse. No difference was observed in the total number of abstinent days and a higher craving was observed in the disulfiram group [47].
	Amitriptyline versus Mirtazapine (1)	56 days	Alcohol craving	The mean alcohol craving scores decreased significantly from baseline to follow-up in both groups. There were no differences in the craving scores between mirtazapine and amitriptyline groups (170.7 SD 26.0 versus 157.7 SD 29.4 at the baseline and 97.3 SD 40.6 versus 99.9 SD40.2 at the endpoint) [27].
	Escitalopram + electroacupuncture (1)	4 weeks	PACS	Zhao and colleagues found that after 4-week treatment, the global scores of PACS declined significantly in both the escitalopram with electroacupuncture and the escitalopram without electroacupuncture groups (both *P* < 0.05). Furthermore, the decline in the rea -electroacupuncture group was superior to that in the sham electroacupuncture group (*P* < 0.05) [96].
Brain stimulation (4)	tDCS (4)	Immediate post treatment 5 weeks 3 and 6 months	Alcohol craving level % of relapse	tDCS significantly decreased alcohol cravings compared to sham stimulation [36,44]. Klauss and colleagues found that the percentage of relapse at 6 months follow-up was higher in the sham group (88%) than the tDCS group (50%) with no difference in cravings between the groups [59]. However, an intensive tDCS scheme was associated with a larger reduction in alcohol cravings when compared to sham-based control, also associated with lower relapse up to 3 months postintervention [60].

AA, Alcoholics Anonymous; FRAMES, Feedback, Responsibility, Advice, Menu, Empathy, Self-efficacy; LMIC, low- and middle-income country; OCDS, Obsessive Compulsive Drinking Scale; PACS, Penn Alcohol Craving Scale; RCT, randomized controlled trial; tDCS, transcranial direct current stimulation.

### Meta-summary

#### Brief interventions

The brief interventions category had the greatest number of RCTs in our study with 24 RCTs, and these interventions were the most similar to each other. The types of interventions included most commonly were WHO-based brief interventions (which utilizes some MI techniques) [28,29,79–82] or MI interventions [22,58,76,88,89,91,93]. Some studies focused more on the intervention delivery, specifically nurse or layperson [67,70,71,75] or computer-based interventions [30,34,35,41]. Outcomes were also varied including harmful alcohol use scores (AUDIT) or alcohol misuse (ASSIST), abstinence or remission (ASSIST), and percent or number of days of drinking or heavy drinking.

Overall, the majority of the studies evaluating brief interventions demonstrated evidence of efficacy in one or more of their alcohol-related outcomes, for both short- (up to 3 months) and long-term (6+ months) outcomes, comparing intervention and control [29,34,35,41, 58,67,70,71,75,76,80,88–90,91,93,94].

WHO-based brief interventions were found to be efficacious to reduce average daily alcohol consumption in males at the health setting [29] and AUDIT average scores in university students [81]. With brief interventions delivered from a motivational intervention framework, Signor and colleagues found at 6-month follow-up, 70% of individuals in the helpline-based brief intervention group and 41% in the control/minimal intervention group had remained abstinent [89]. Similarly, this mode of delivery showed evidence of efficacy at the primary care setting to reduce alcohol use [76,91,93] and with university students [58]. Simao found that college students had a significant reduction in the amount of alcohol use per occasion and AUDIT scores up to 24 months after the intervention [90]. Other modes of delivery revealed that lay counselor–delivered interventions had significant differences between intervention and control [67,71,75], and a computerized intervention reduced alcohol use as much as an in-person motivational intervention [41]. One study focused on the efficacy of a women-focused brief intervention demonstrated efficacy of the interventions in reducing heavy drinking behavior and heavy drinking days in women [94].

However, there were some studies that found a similar reduction in alcohol-related outcomes between both the intervention and control groups, thus a null effect [22,28,30,79,80,82,88]. Assangkornchai and Pengpid found brief interventions at the primary care setting addressing alcohol and other substances reduced alcohol use for both intervention and control arms equally [28,80,82]. Similarly, those evaluating a brief intervention in tuberculosis patients in the inpatient or outpatient setting showed limited results [22,79]. Web-based, personalized normative feedback (PNF) interventions showed conflicting results in 2 studies with similar populations [30,34]. Nadkarni and colleagues found a reduction in alcohol consumed and mean AUDIT score for both MI-based interventions but no difference between the groups; however, this feasibility study was not powered to detect such differences [70,72].

#### Psychotherapy or counseling

Overall, 15 RCTs matched our definition of psychotherapy or counseling. Interventions in this group varied in terms of length, population, and framework. Most commonly, interventions used MI techniques [22,23,61,69,72,83] or cognitive behavioral therapy (CBT) [53,64,68,77,78,87]. Some interventions used education and stigma reduction [73] or a combination of methods [45,92]. These studies also had varied populations, including hospitalized patients [45,63,73,74,87,91], emergency department patients [92], outpatient primary care patients [69], and patients visiting clinics specializing in reproductive health [61,83], HIV or tuberculosis [22,77], and substance abuse [23,64].

A number of the studies found a reduction in alcohol-related outcomes. Daengthoen’s intensive inpatient rehabilitation combination therapy intervention had a reduction in alcohol consumption and drink cravings [45]. Nattala found that a significantly higher percentage of dyadic intervention patients (57%) were abstinent compared to individual treatment (27%) or treatment as usual (30%) patients [73]. Sorsdahl and colleagues found a reduction in the ASSIST scale for those who received the MI with problem solving intervention compared to the control, yet there was no difference between the MI alone and the control group [92]. L’Engle, Rendall-Mkosi, and Moraes all had significant findings for their MI interventions reducing binge drinking up to 12 months, reducing the proportion of women at risk for alcohol-exposed pregnancies and increasing the proportion of abstinent patients [23,61,83]. Ng and colleagues used a body–mind–spirit multidimensional intervention and reported significantly less alcohol cravings, drinking days, drinks per drinking day, and relapse in the intervention group compared to treatment as usual at 3 months [74]. Randomization to receive CBT, in different modalities, was found to be associated with a higher reduction in drinking days, drinks per drinking days [77,78], and AUDIT score [63] in comparison to usual care, at 3 months for HIV-infected outpatients reduction in mean AUDIT score [68], and alcohol consumption [53] in participants positive for intimate violence.

A few of the studies in this subgroup had null effects or found no difference between the intervention and control arms. Marques and colleagues found a reduction in many of their alcohol-related outcomes for the group and individual intervention arms at 15 months, but the intervention arms were not significantly different from each other [64]. Similarly, Satyanarayan found a reduction in Severity of Alcohol Dependence Questionnaire (SADQ) scores for CBT and usual care arm patients but no significant difference between intervention arms; authors believed that this was because both intervention arms received similar alcohol reduction strategy intervention components [87]. Alternatively, Shin and colleagues found that their intervention, which focused on inpatient tuberculosis patients with severe AUDs, caused no change in alcohol-related outcomes, likely because the study did not include alcohol treatment–seeking patients, but had patients with low readiness to change or poor intervention participation rates combined with relatively low enrollment numbers [22].

#### Health promotion and education

In total, we found 20 RCTs, which evaluated health promotion and education interventions. Of these, 2 were based in the workplace [26,38], 5 in the community [23,37,43,84], 6 in schools [33,40,65,85,86], and 7 in clinics [39,55–57], of which 1 was focused on women sex workers [95]. The majority of programs addressed alcohol use in the context of HIV/AIDS prevention and risk reduction [38–40,43,56,57,95].

About half (8 of 17) of the health promotion and education interventions were found to have positive results [25,26,38,39,54,56,57,65,66,84]. Some of these studies also had mixed results. For example, Aira and colleagues found a reduction in drinks per day and an improvement in attitudes toward drinking, but not a reduction in the total amount of alcohol consumption [26]. Similarly, Rotheram-Borus found that home visits for pre- and postnatal women were associated with a reduction in the use of alcohol during pregnancy, but this drinking resumed postpartum [84].

Meanwhile, a majority of the studies that found no effect of their interventions either were not adequately powered to detect the alcohol-related outcome [23,43] or were compared to another intervention rather than a control, thus potentially obscuring some potential reduction in harm [95]. Cubbins and colleagues evaluated a community-level intervention in which popular community individuals relayed education through casual conversations and found significant alcohol reduction in both the intervention and control groups, but no difference between the groups [43]. Chhabra and colleagues looked at the effectiveness of a Severity of Alcohol Dependence Questionnaire (STEP) school-based program but found that students, and more specifically girls, had an immediate reduction in their intent to use alcohol, but there was no difference in the intention to use alcohol at the 10-week outcome assessment [40].

#### Biomedical treatments

The final group of RCTs evaluated biomedical treatments and included 19 RCTs evaluating medications and brain stimulation. The 14 RCTs evaluating medications looked at naltrexone (3) [22,24,32], ondansetron (1) [43], gabapentin (1) [51], disulfiram (5) [46–50], and topiramate (2) [32,62]. Two RCTs evaluated combined behavioral and medication interventions: evaluated acamprosate with Alcoholics Anonymous (AA) [31] and one evaluated baclofen with a brief intervention [52]. One RCT evaluated the efficacy of adding acupuncture to an escitalopram treatment regimen [96].

The RCTs evaluating naltrexone and ondansetron found limited impact on abstinence, number of heavy drinking days or number of abstinent days [22,24,32], and abstinence [43]. Mixed effects were found by RCTs for topiramate, where Baltieri found an increase in abstinence at 4 weeks, although Likhitsathian found no differences in any drinking quantity or frequency measures up to 12 weeks [32,62].

On the other hand, the RCTs evaluating gabapentin, acamprosate, and baclofen exhibit more positive results. Furieri found that gabapentin was associated with a significant decrease in quantity and frequency of drinking and higher mean abstinent days [51]. Baltieri found that acamprosate improved abstinence rates but only for those who participated in AA [31]. Moreover, Gupta found that patients who received baclofen compared to a nutritional supplement, with a brief motivational intervention, were more likely to remain abstinent, have lower heavy drinking days, and fewer alcohol cravings [52].

We identified 4 RCTs that studied transcranial direct current stimulation, and all of them occurred at 2 institutions in Brazil. While 3 of these studies found a decrease in alcohol cravings compared to sham stimulation [36,44,60], one study found a lower relapse rate in the brain stimulation group but with no difference in alcohol cravings at 6-month follow-up [59]. Ultimately, 3 of the 4 studies in this group found more relapses in the intervention group at 4-week, 6-month, and 12-month follow-up [44,59,60].

## Discussion

This is the first review, to our knowledge, of alcohol harm reduction interventions evaluated in LMICs. Most studies we found took place in middle-income countries; there was a noticeable gap of studies in the Middle East, North Africa, Europe, Central Asia, and South Asia regions. Overall, we found that there was limited uniformity for interventions, outcomes, and follow-up times across studies, which limited our ability to compare results. The vast majority of evaluations were limited to middle-income settings, leading to feasibility and generalizability concerns for low-income settings. Of all the RCTs, brief interventions were the most commonly studied; similarly, MI techniques were the most prevalent behavior change technique common in both brief and psychotherapy and counseling interventions. Brief interventions and motivational interviewing techniques also had the most consistent positive results in our findings.

### Lack of uniformity limits effective comparisons

The studies included in our meta-summary used a wide variety of metrics to measure alcohol-related outcomes of alcohol interventions; these metrics included (i) AUDIT scores; (ii) ASSIST scores; (iii) # of drinking days; (iv) # heavy drinking days; (v) # drinks per drinking day; (vi) # abstinent days; (vii) # drinks per day; (viii) % of patients abstinent; and (ix) % of patients relapsing.

The time period over which these outcomes were measured also varied considerably, from 3 months [41] to 24 months [90]. This lack of uniformity compromised our ability to discern the effectiveness of interventions or to compare results across studies. The diversity of alcohol consumption outcomes measures is due in part to varying recall, reference period, and definition of a “standard drink” [97–99]. Future study studies may benefit from using consistent outcome measures and adopting uniform methods of intervention implementation or study designs.

### Uncertain feasibility of implementing interventions in low-income country setting

The vast majority of the studies in this review were conducted in middle-income countries. Thus, the feasibility of implementing these interventions and their effectiveness in low-income settings is uncertain. Low-income countries face greater barriers (such as scarcity of medical facilities, limited training available to medical staff, infrastructural barriers to healthcare access, and effective patient communication/follow-up) to implementing effective healthcare than either high- or middle-income countries. As a clear example, all 4 studies [36,44,59,60] that used brain stimulation as an intervention were conducted in Brazil, an upper middle-income country. In addition to its uncertain effectiveness, brain stimulation requires expensive equipment and specific facilities, and it is not likely to be feasible in some low-income country settings. In another example, although psychotherapy and counseling interventions are demonstrably effective [45,61,73,83,92], none of these studies took place in a low-income country, so the feasibility of implementing this type of intervention is uncertain. Given that infrastructure in low-income settings is even more limited in mental health and substance abuse facilities and professionals, with a greater associated stigma, an alcohol use reduction intervention implementation of this kind is still potentially unfeasible. Similarly, medication shows some evidence of a positive effect on abstinence from alcohol, but reliable availability of medication is essential for this intervention to be effective; thus, medication may not be a feasible intervention in a low-income country [100,101].

Instead, the most studied and potentially most feasible intervention is a brief intervention. In our systematic review, 6 studies evaluated brief interventions in South Africa [67,79–81,93,94]. Brief interventions have been studied to decrease alcohol use and alcohol-related consequences in a variety of settings and countries [102–105]. They have also been suggested to be cost-effective in high-income countries [106]. In low- and middle-income settings, brief interventions are likely to be feasible because they can be delivered by nonprofessionals requiring less training.

## Limitations

There are a few limitations to our study. First, our search strategy did not exclude studies due to language, and, yet, we found no manuscripts in other languages. Thus, either there is no non-English language literature on this topic or the data sets we searched have limited non-English language articles. Second, our ability to conduct a thorough meta-analysis was restricted by nonuniform outcome measurements, a wide variety of outcome assessment times, and a wide variety of interventions, making it difficult to compare interventions and their effects. To compensate for this, we summarized results from RCTs qualitatively. Similarly, we conducted our database search for only LMICs at that time. This might limit our findings by excluding articles from countries that have become high income since the study occurred or erroneously including countries that were high income but then reduced their status at the time of the database search; in the former case, we cannot determine the number of potential studies, but for the latter case, we rechecked the World Bank status of all countries and their intervention time periods to ensure this was not occurring. Finally, we tried to group types of interventions based on standard definitions rather than study-specific descriptions that might limit the interpretation of effect size and differ from the original author’s description.

## Improving future research

Future alcohol harm reduction intervention studies should use uniform reporting. Studies ranged widely in their intervention type, framework, population, augmentation, or boosters, as well as outcome assessment frequency and timing. Adherence to 1 or 2 sets of standardized outcome reporting measures at a specified time period would greatly improve comparability across time and geographic location, allowing for a meta-analysis of intervention methods. Based on our review, brief interventions using the ASSIST or AUDIT scoring systems are the most widely used and appear to provide the best standardization among outcomes. Overall, future research should include both comparative effectiveness to determine best interventions for LMIC settings but also most effective implementation strategies including target populations.

## Conclusions

In conclusion, alcohol harm reduction interventions in LMICs are nonuniform in nature, skewed in geographic regions where applied, and result in uncertain effectiveness over varying time horizons. Feasible options specific to low-income countries are most likely brief interventions and interventions that utilize motivational interviewing techniques. Identifying uniform methods of implementation and assessment of alcohol harm reduction interventions can be a first step toward establishing a set of evidence-based protocols for treatment for low-income settings. Current studies in brief interventions, psychotherapy, and brain stimulation show promise, but have been tested primarily or exclusively in middle-income settings. Feasibility testing in low-income settings, comparative effectiveness testing, and uniform reporting methods are needed to help determine the most effective alcohol harm reduction strategies for low-income settings in order to address this global health crisis.

## Supporting information

S1 Fig Search strategy. (DOCX)

S2 Fig Map of study location and intervention type. Source: Global Administrative Areas (2022). University of California, Berkely. Available online: http://www.gadm.org [11/03/2022]; https://geodata.ucdavis.edu/gadm/gadm4.0/gadm404-shp.zip.
(TIF)

S1 Table PRISMA Checklist. PRISMA, Preferred Reporting Items for Systematic Reviews and Meta-Analyses.
(DOCX)

## Abbreviations

AA: Alcoholics Anonymous
ACROBAT-NRS: A Cochrane Risk Of Bias Assessment Tool for Non-Randomized Studies
ASSIST: Alcohol, Smoking and Substance Involvement Screening Test
AUD: alcohol use disorder
AUDIT: Alcohol Use Disorders Identification Test
CBT: cognitive behavioral therapy
DALY: disability-adjusted life year
LMIC: low- and middle-income country
MI: motivational interviewing
NOS: Newcastle–Ottawa scale
PNF: personalized normative feedback
PRISMA: Preferred Reporting Items for Systematic Reviews and Meta-Analyses
RAPI: Rutgers Alcohol Problem Index
RCT: randomized controlled trial
SADQ: Severity of Alcohol Dependence Questionnaire
STEP: School-based Teenage Education Program
STROBE: STrengthening the Reporting of OBservational studies in Epidemiology
WHO: World Health Organization
