# Supplementary material for: Patient-level interventions to reduce alcohol-related harms in low- and middle-income countries: A systematic review and meta-summary
Source: PLoS Med. 2022 Apr 12;19(4):e1003961. doi: 10.1371/journal.pmed.1003961 (PMC9004752; doi:10.1371/journal.pmed.1003961)
Supplement: S1 Fig — (DOCX) [file pmed.1003961.s001.docx]

PubMed Search Strategy

| Set | Terms | Results |
| --- | --- | --- |
| #1 | "Alcohol-Related Disorders"[Mesh] OR "Alcoholic Beverages"[Mesh] OR "Alcohol Drinking"[Mesh] OR ((heavy[tiab] OR hazardous[tiab] OR harmful[tiab] OR excessive[tiab] OR problem[tiab] OR binge[tiab] OR controlled[tiab] OR risky[tiab] OR "at risk"[tiab] OR "at-risk"[tiab] OR use[tiab]) AND alcohol[tiab] AND drink*[tiab]) OR alcoholism[tiab] | 166614 |
| #2 | "Alcohol-Related Disorders/therapy"[Mesh] OR "Alcoholic Beverages/legislation and jurisprudence"[Mesh] OR "Psychotherapy"[Mesh] OR "Counseling"[Mesh] OR "Health Education"[Mesh] OR "Motivational Interviewing"[Mesh] OR "Advertising as Topic"[Mesh] OR "Mass Media"[Mesh] OR "Telecommunications"[Mesh] OR "Public Policy"[Mesh] OR "Telemedicine"[Mesh] OR "Mobile Applications"[Mesh] OR "Cell Phones"[Mesh] OR "Text Messaging"[Mesh] OR "Computers, Handheld"[Mesh] OR "Community Mental Health Services"[Mesh] OR Intervention[tiab] OR interventions[tiab] OR "motivational interviewing" OR psychotherapy[tiab] OR counseling[tiab] OR "cognitive therapy"[tiab] OR "behavior therapy"[tiab] OR "behaviour therapy"[tiab] OR "behavioural therapy"[tiab] OR "behavioral therapy"[tiab] OR advertis*[tiab] OR billboard*[tiab] OR broadcast*[tiab] OR radio[tiab] OR telecommunications[tiab] OR media[tiab] OR policy[tiab] OR "mobile applications"[tiab] OR "mobile application"[tiab] OR cellphone[tiab] OR cellphones[tiab] OR ((mobile[tiab] OR cellular[tiab] OR cell[tiab]) AND (phone[tiab] OR phones[tiab] OR device[tiab] OR devices[tiab] OR app[tiab] OR apps[tiab])) OR mobiles[tiab] OR smartphone[tiab] OR smartphones[tiab] OR telephone[tiab] OR telephones[tiab] OR phone[tiab] OR phones[tiab] OR e-health[tiab] OR ehealth[tiab] OR m-health [tiab] OR mhealth[tiab] OR telehealth[tiab] OR telemedicine[tiab] OR video-conference[tiab] OR videoconference[tiab] OR video-conferencing[tiab] OR videoconferencing[tiab] OR "automated telephone"[tiab] OR IVR[tiab] OR "interactive voice response"[tiab] OR sms[tiab] OR skype[tiab] OR (text[tiab] AND (message[tiab] OR messages[tiab])) OR texts[tiab] OR mms[tiab] OR blackberry[tiab] OR ipad[tiab] OR ipads[tiab] OR android[tiab] OR laptop[tiab] OR laptops[tiab] OR ((tablet[tiab] OR tablets[tiab]) AND (computer[tiab] OR computers[tiab] OR device[tiab] OR devices[tiab])) OR ((family[tiab] OR community[tiab] OR school*[tiab] OR "Schools"[Mesh] OR "Family"[Mesh] OR psychosocial[tiab] OR education[tiab]) AND (program*[tiab] OR intervention*[tiab])) | 1787525 |
| #3 | "Developing Countries"[Mesh] OR "Afghanistan"[Mesh] OR "Bangladesh"[Mesh] OR "Benin"[Mesh] OR "Burkina Faso"[Mesh] OR "Burundi"[Mesh] OR "Cambodia"[Mesh] OR "Central African Republic"[Mesh] OR "Chad"[Mesh] OR "Comoros"[Mesh] OR "Democratic Republic of the Congo"[Mesh] OR "Eritrea"[Mesh] OR "Ethiopia"[Mesh] OR "Gambia"[Mesh] OR "Guinea"[Mesh] OR "Guinea-Bissau"[Mesh] OR "Haiti"[Mesh] OR "Kenya"[Mesh] OR "Democratic People's Republic of Korea"[Mesh] OR "Liberia"[Mesh] OR "Madagascar"[Mesh] OR "Malawi"[Mesh] OR "Mali"[Mesh] OR "Mozambique"[Mesh] OR "Myanmar"[Mesh] OR "Nepal"[Mesh] OR "Niger"[Mesh] OR "Rwanda"[Mesh] OR "Sierra Leone"[Mesh] OR "Somalia"[Mesh] OR "Tajikistan"[Mesh] OR "Tanzania"[Mesh] OR "Togo"[Mesh] OR "Uganda"[Mesh] OR "Zimbabwe"[Mesh] OR "Armenia"[Mesh] OR "Bhutan"[Mesh] OR "Bolivia"[Mesh] OR "Cameroon"[Mesh] OR "Cape Verde"[Mesh] OR "Congo"[Mesh] OR "Cote d'Ivoire"[Mesh] OR "Djibouti"[Mesh] OR "Egypt"[Mesh] OR "El Salvador"[Mesh] OR "Georgia (Republic)"[Mesh] OR "Ghana"[Mesh] OR "Guatemala"[Mesh] OR "Guyana"[Mesh] OR "Honduras"[Mesh] OR "Indonesia"[Mesh] OR "India"[Mesh] OR "Kosovo"[Mesh] OR "Kyrgyzstan"[Mesh] OR "Laos"[Mesh] OR "Lesotho"[Mesh] OR "Mauritania"[Mesh] OR "Micronesia"[Mesh] OR "Moldova"[Mesh] OR "Mongolia"[Mesh] OR "Morocco"[Mesh] OR "Nicaragua"[Mesh] OR "Nigeria"[Mesh] OR "Pakistan"[Mesh] OR "Papua New Guinea"[Mesh] OR "Paraguay"[Mesh] OR "Philippines"[Mesh] OR "Independent State of Samoa"[Mesh] OR "Atlantic Islands"[Mesh] OR "Senegal"[Mesh] OR "Melanesia"[Mesh] OR "Sri Lanka"[Mesh] OR "Sudan"[Mesh] OR "Swaziland"[Mesh] OR "Syria"[Mesh] OR "Timor-Leste"[Mesh] OR "Ukraine"[Mesh] OR "Uzbekistan"[Mesh] OR "Vanuatu"[Mesh] OR "Vietnam"[Mesh] OR "Middle East"[Mesh] OR "Yemen"[Mesh] OR "Zambia"[Mesh] OR "Angola"[Mesh] OR "Albania"[Mesh] OR "Algeria"[Mesh] OR "American Samoa"[Mesh] OR "Argentina"[Mesh] OR "Azerbaijan"[Mesh] OR "Republic of Belarus"[Mesh] OR "Belize"[Mesh] OR "Bosnia and Herzegovina"[Mesh] OR "Botswana"[Mesh] OR "Brazil"[Mesh] OR "Bulgaria"[Mesh] OR "China"[Mesh] OR "Colombia"[Mesh] OR "Costa Rica"[Mesh] OR "Cuba"[Mesh] OR "Dominica"[Mesh] OR "Dominican Republic"[Mesh] OR "Ecuador"[Mesh] OR "Equatorial Guinea"[Mesh] OR "Fiji"[Mesh] OR "Gabon"[Mesh] OR "Grenada"[Mesh] OR "Iran"[Mesh] OR "Iraq"[Mesh] OR "Jamaica"[Mesh] OR "Jordan"[Mesh] OR "Kazakhstan"[Mesh] OR "Lebanon"[Mesh] OR "Libya"[Mesh] OR "Macedonia (Republic)"[Mesh] OR "Malaysia"[Mesh] OR "Indian Ocean Islands"[Mesh] OR "Mexico"[Mesh] OR "Montenegro"[Mesh] OR "Namibia"[Mesh] OR "Palau"[Mesh] OR "Panama"[Mesh] OR "Peru"[Mesh] OR "Romania"[Mesh] OR "Russia"[Mesh] OR "Serbia"[Mesh] OR "Seychelles"[Mesh] OR "South Africa"[Mesh] OR "Saint Lucia"[Mesh] OR "Saint Vincent and the Grenadines"[Mesh] OR "Suriname"[Mesh] OR "Thailand"[Mesh] OR "Tonga"[Mesh] OR "Tunisia"[Mesh] OR "Turkey"[Mesh] OR "Turkmenistan"[Mesh] OR "Venezuela"[Mesh] OR "Afghanistan"[all fields] OR "Bangladesh"[all fields] OR "Benin"[all fields] OR "Burkina Faso"[all fields] OR "Burundi"[all fields] OR "Cambodia"[all fields] OR "cabo verde"[all fields] OR "Central African Republic"[all fields] OR "Chad"[all fields] OR "Comoros"[all fields] OR "Democratic Republic of the Congo"[all fields] OR "Eritrea"[all fields] OR "Ethiopia"[all fields] OR "Gambia"[all fields] OR "Guinea"[all fields] OR "Guinea-Bissau"[all fields] OR "Haiti"[all fields] OR "Kenya"[all fields] OR "Democratic People's Republic of Korea"[all fields] OR "Liberia"[all fields] OR "Madagascar"[all fields] OR "Malawi"[all fields] OR "Mali"[all fields] OR "Mozambique"[all fields] OR "Myanmar"[all fields] OR "Nepal"[all fields] OR "Niger"[all fields] OR "Rwanda"[all fields] OR "Sierra Leone"[all fields] OR "Somalia"[all fields] OR "Tajikistan"[all fields] OR "Tanzania"[all fields] OR "Togo"[all fields] OR "Uganda"[all fields] OR "Zimbabwe"[all fields] OR "Armenia"[all fields] OR "Bhutan"[all fields] OR "Bolivia"[all fields] OR "Cameroon"[all fields] OR "Cape Verde"[all fields] OR "Congo"[all fields] OR "Cote d'Ivoire"[all fields] OR "Djibouti"[all fields] OR "Egypt"[all fields] OR "El Salvador"[all fields] OR "Georgia (Republic)"[all fields] OR "Ghana"[all fields] OR "Guatemala"[all fields] OR "Guyana"[all fields] OR "Honduras"[all fields] OR "Indonesia"[all fields] OR "India"[all fields] OR “Kiribati”[all fields] OR "Kosovo"[all fields] OR "Kyrgyzstan"[all fields] OR "Kyrgyz"[all fields] OR "Laos"[all fields] OR "lao"[all fields] OR "Lesotho"[all fields] OR "Mauritania"[all fields] OR "Micronesia"[all fields] OR "Moldova"[all fields] OR "Mongolia"[all fields] OR "Morocco"[all fields] OR "Nicaragua"[all fields] OR "Nigeria"[all fields] OR "Pakistan"[all fields] OR "Papua New Guinea"[all fields] OR "Paraguay"[all fields] OR "Philippines"[all fields] OR "Independent State of Samoa"[all fields] OR "Atlantic Islands"[all fields] OR "Sao Tome"[all fields] OR Principe[all fields] OR "Senegal"[all fields] OR "Melanesia"[all fields] OR "Solomon islands"[all fields] OR "Sri Lanka"[all fields] OR "Sudan"[all fields] OR "Swaziland"[all fields] OR "Syria"[all fields] OR "East Timor"[all fields] OR "Timor leste"[all fields] OR "Ukraine"[all fields] OR "Uzbekistan"[all fields] OR "Vanuatu"[all fields] OR "Vietnam"[all fields] OR "Middle East"[all fields] OR "west bank"[all fields] OR "Gaza"[all fields] OR "Yemen"[all fields] OR "Zambia"[all fields] OR "Angola"[all fields] OR "Albania"[all fields] OR "Algeria"[all fields] OR "Samoa"[all fields] OR "Azerbaijan"[all fields] OR "Republic of Belarus"[all fields] OR "Belize"[all fields] OR "Bosnia-Herzegovina"[all fields] OR "Botswana"[all fields] OR "Brazil"[all fields] OR "Bulgaria"[all fields] OR "China"[all fields] OR "Colombia"[all fields] OR "Costa Rica"[all fields] OR "Cuba"[all fields] OR "Dominica"[all fields] OR "Dominican Republic"[all fields] OR "Ecuador"[all fields] OR "Fiji"[all fields] OR "Gabon"[all fields] OR "Grenada"[all fields] OR "Iran"[all fields] OR "Iraq"[all fields] OR "Jamaica"[all fields] OR "Jordan"[all fields] OR "Kazakhstan"[all fields] OR "Lebanon"[all fields] OR "Libya"[all fields] OR "Macedonia"[all fields] OR "Malaysia"[all fields] OR "Indian Ocean Islands"[all fields] OR "Maldives"[all fields] OR “Marshall Islands”[all fields] OR "Mauritius"[all fields] OR "Mexico"[all fields] OR "Montenegro"[all fields] OR "Namibia"[all fields] OR "Palau"[all fields] OR "Panama"[all fields] OR "Peru"[all fields] OR "Romania"[all fields] OR "Serbia"[all fields] OR "Seychelles"[all fields] OR "South Africa"[all fields] OR "Saint Lucia"[all fields] OR "Saint Vincent and the Grenadines"[all fields] OR "Suriname"[all fields] OR "Thailand"[all fields] OR "Tonga"[all fields] OR "Tunisia"[all fields] OR "Turkey"[all fields] OR "Turkmenistan"[all fields] OR "Tuvalu"[all fields] OR "low resource"[all fields] OR "under-resourced"[all fields] OR "resource poor"[all fields] OR "under-developed"[all fields] OR "underdeveloped"[all fields] OR "developing country"[all fields] OR "developing countries"[all fields] OR "developing world"[all fields] OR "third world" [all fields] OR lmic[all fields] OR (low[all fields] AND middle[all fields] AND income[all fields]) | 3204583 |
| #4 | #1 AND #2 AND #3 | 3718 |
| #5 | (randomized controlled trial[pt] OR controlled clinical trial[pt] OR randomized[tiab] OR randomised[tiab] OR randomization[tiab] OR randomisation[tiab] OR randomly[tiab] OR trial[tiab] OR groups[tiab] OR Nonrandom[tiab] OR non-random[tiab] OR nonrandomized[tiab] OR non-randomized[tiab] OR nonrandomized[tiab] OR non-randomised[tiab] OR quasi-experiment*[tiab] OR quasiexperiment*[tiab] OR quasirandom*[tiab] OR quasi-random*[tiab] OR quasi-control*[tiab] OR quasicontrol*[tiab] OR (controlled[tiab] AND (trial[tiab] OR study[tiab])) OR Clinical trial[pt] OR “clinical trial”[tiab] OR “clinical trials”[tiab] OR "evaluation studies"[Publication Type] OR "evaluation studies as topic"[MeSH] OR "evaluation study"[tiab] OR evaluation studies[tiab] OR "intervention study"[tiab] OR "intervention studies"[tiab] OR "case-control studies"[MeSH] OR "case-control"[tiab] OR "cohort studies"[MeSH] OR cohort[tiab] OR "longitudinal studies"[MeSH] OR "longitudinal”[tiab] OR longitudinally[tiab] OR "prospective"[tiab] OR prospectively[tiab] OR "retrospective studies"[MeSH] OR "retrospective"[tiab] OR "follow up"[tiab] OR "comparative study"[pt] OR "comparative study"[tiab] OR systematic[subset] OR "meta-analysis"[pt] OR "meta-analysis as topic"[MeSH] OR "meta-analysis"[tiab] OR "meta-analyses"[tiab]) NOT (Editorial[pt] OR Letter[pt] OR Case Reports[pt] OR Comment[pt]) NOT (animals[mh] NOT humans[mh]) | 5386535 |
| #6 | #4 AND #5 | 1602 |

Embase

| Set | Terms | Results |
| --- | --- | --- |
| #1 | 'alcoholism'/exp OR 'alcoholic beverage'/exp OR 'drinking behavior'/exp OR alcohol:ab,ti OR alcoholism:ab,ti OR ((heavy:ab,ti OR hazardous:ab,ti OR harmful:ab,ti OR excessive:ab,ti OR problem:ab,ti OR binge:ab,ti OR controlled:ab,ti OR risky:ab,ti OR "at risk":ab,ti OR "at-risk":ab,ti OR use:ab,ti) AND alcohol:ab,ti AND drink*:ab,ti) | 380837 |
|  | 'alcoholism'/exp/dm_th OR 'psychotherapy'/exp OR 'counseling'/exp OR 'health education'/exp OR 'motivational interviewing'/exp OR 'advertising'/exp OR 'mass communication'/exp OR 'policy'/exp OR 'telehealth'/exp OR 'mobile application'/exp OR 'mobile phone'/exp OR 'text messaging'/exp OR 'personal digital assistant'/exp OR 'mental health service'/exp OR Intervention:ab,ti OR interventions:ab,ti OR "motivational interviewing" OR psychotherapy:ab,ti OR counseling:ab,ti OR "cognitive therapy":ab,ti OR "behavior therapy":ab,ti OR "behaviour therapy":ab,ti OR "behavioural therapy":ab,ti OR "behavioral therapy":ab,ti OR advertis*:ab,ti OR billboard*:ab,ti OR broadcast*:ab,ti OR radio:ab,ti OR telecommunications:ab,ti OR media:ab,ti OR policy:ab,ti OR "mobile applications":ab,ti OR "mobile application":ab,ti OR cellphone:ab,ti OR cellphones:ab,ti OR ((mobile:ab,ti OR cellular:ab,ti OR cell:ab,ti) AND (phone:ab,ti OR phones:ab,ti OR device:ab,ti OR devices:ab,ti OR app:ab,ti OR apps:ab,ti)) OR mobiles:ab,ti OR smartphone:ab,ti OR smartphones:ab,ti OR telephone:ab,ti OR telephones:ab,ti OR phone:ab,ti OR phones:ab,ti OR 'e health':ab,ti OR ehealth:ab,ti OR 'm health':ab,ti OR mhealth:ab,ti OR telehealth:ab,ti OR telemedicine:ab,ti OR 'video conference':ab,ti OR videoconference:ab,ti OR 'video conferencing':ab,ti OR videoconferencing:ab,ti OR "automated telephone":ab,ti OR IVR:ab,ti OR "interactive voice response":ab,ti OR sms:ab,ti OR skype:ab,ti OR (text:ab,ti AND (message:ab,ti OR messages:ab,ti)) OR texts:ab,ti OR mms:ab,ti OR blackberry:ab,ti OR ipad:ab,ti OR ipads:ab,ti OR android:ab,ti OR laptop:ab,ti OR laptops:ab,ti OR ((tablet:ab,ti OR tablets:ab,ti) AND (computer:ab,ti OR computers:ab,ti OR device:ab,ti OR devices:ab,ti)) OR ((psychosocial:ab,ti OR education:ab,ti OR 'school'/exp OR 'family'/exp OR family:ab,ti OR community:ab,ti OR school*:ab,ti) AND (program*:ab,ti OR intervention*:ab,ti)) | 2718275 |
|  | 'developing country'/exp OR 'Afghanistan'/exp OR 'Bangladesh'/exp OR 'Benin'/exp OR 'Burkina Faso'/exp OR 'Burundi'/exp OR 'Cambodia'/exp OR 'Central African Republic'/exp OR 'Chad'/exp OR 'Comoros'/exp OR 'Democratic Republic Congo'/exp OR 'Congo'/exp OR 'Eritrea'/exp OR 'Ethiopia'/exp OR 'Gambia'/exp OR 'Guinea'/exp OR 'Guinea-Bissau'/exp OR 'Haiti'/exp OR 'Kenya'/exp OR 'North Korea'/exp OR 'Liberia'/exp OR 'Madagascar'/exp OR 'Malawi'/exp OR 'Mozambique'/exp OR 'Myanmar'/exp OR 'Nepal'/exp OR 'Niger'/exp OR 'Nigeria'/exp OR 'Rwanda'/exp OR 'Sierra Leone'/exp OR 'Somalia'/exp OR 'Tajikistan'/exp OR 'Tanzania'/exp OR 'Togo'/exp OR 'Uganda'/exp OR 'Zimbabwe'/exp OR 'Armenia'/exp OR 'Bhutan'/exp OR 'Bolivia'/exp OR 'Cameroon'/exp OR 'Cape Verde'/exp OR 'Cote d`Ivoire'/exp OR 'Djibouti'/exp OR 'Egypt'/exp OR 'El Salvador'/exp OR 'Georgia (republic)'/exp OR 'Ghana'/exp OR 'Guatemala'/exp OR 'Guyana'/exp OR 'Honduras'/exp OR 'Indonesia'/exp OR 'India'/exp OR 'Kosovo'/exp OR 'Kyrgyzstan'/exp OR 'Laos'/exp OR 'Lesotho'/exp OR 'Mauritania'/exp OR 'Federated States of Micronesia'/exp OR 'Moldova'/exp OR 'Mongolia'/exp OR 'Nicaragua'/exp OR 'Pakistan'/exp OR 'Papua New Guinea'/exp OR 'Philippines'/exp OR 'Samoa'/exp OR 'Sao Tome and Principe'/exp OR 'Senegal'/exp OR 'Solomon Islands'/exp OR 'Sri Lanka'/exp OR 'Sudan'/exp OR 'Swaziland'/exp OR 'Syrian Arab Republic'/exp OR 'Timor-Leste'/exp OR 'Ukraine'/exp OR 'Uzbekistan'/exp OR 'Vanuatu'/exp OR 'Viet Nam'/exp OR 'Yemen'/exp OR 'Zambia'/exp OR 'Angola'/exp OR 'Albania'/exp OR 'Algeria'/exp OR 'American Samoa'/exp OR 'Argentina'/exp OR 'Azerbaijan'/exp OR 'Belarus'/exp OR 'Belize'/exp OR 'Bosnia and Herzegovina'/exp OR 'Botswana'/exp OR 'Brazil'/exp OR 'Bulgaria'/exp OR 'China'/exp OR 'Colombia'/exp OR 'Costa Rica'/exp OR 'Cuba'/exp OR 'Dominica'/exp OR 'Dominican Republic'/exp OR 'Ecuador'/exp OR 'Equatorial Guinea'/exp OR 'Fiji'/exp OR 'Gabon'/exp OR 'Grenada'/exp OR 'Iran'/exp OR 'Iraq'/exp OR 'Jamaica'/exp OR 'Jordan'/exp OR 'Kazakhstan'/exp OR 'Lebanon'/exp OR 'Libyan Arab Jamahiriya'/exp OR 'Macedonia (republic)'/exp OR 'Malaysia'/exp OR 'Maldives'/exp OR 'Mexico'/exp OR 'Montenegro (republic)'/exp OR 'Namibia'/exp OR 'Palau'/exp OR 'Panama'/exp OR 'Peru'/exp OR 'Romania'/exp OR 'Russian Federation'/exp OR 'Serbia'/exp OR 'Seychelles'/exp OR 'South Africa'/exp OR 'Saint Lucia'/exp OR 'Saint Vincent and the Grenadines'/exp OR 'Suriname'/exp OR 'Thailand'/exp OR 'Tonga'/exp OR 'Tunisia'/exp OR 'Turkey (republic)'/exp OR 'Turkmenistan'/exp OR 'Venezuela'/exp OR 'Afghanistan':ab,ti,ca OR 'Bangladesh':ab,ti,ca OR 'Benin':ab,ti,ca OR 'Burkina Faso':ab,ti,ca OR 'Burundi':ab,ti,ca OR 'Cambodia':ab,ti,ca OR 'cabo verde':ab,ti,ca OR 'Central African Republic':ab,ti,ca OR 'Chad':ab,ti,ca OR 'Comoros':ab,ti,ca OR 'Congo':ab,ti,ca OR 'Eritrea':ab,ti,ca OR 'Ethiopia':ab,ti,ca OR 'Gambia':ab,ti,ca OR 'Guinea':ab,ti,ca OR 'Haiti':ab,ti,ca OR 'Kenya':ab,ti,ca OR 'Korea':ab,ti,ca OR 'Liberia':ab,ti,ca OR 'Madagascar':ab,ti,ca OR 'Malawi':ab,ti,ca OR 'Mali':ab,ti,ca OR 'Mozambique':ab,ti,ca OR 'Myanmar':ab,ti,ca OR 'Nepal':ab,ti,ca OR 'Niger':ab,ti,ca OR 'Rwanda':ab,ti,ca OR 'Sierra Leone':ab,ti,ca OR 'Somalia':ab,ti,ca OR 'Tajikistan':ab,ti,ca OR 'Tanzania':ab,ti,ca OR 'Togo':ab,ti,ca OR 'Uganda':ab,ti,ca OR 'Zimbabwe':ab,ti,ca OR 'Armenia':ab,ti,ca OR 'Bhutan':ab,ti,ca OR 'Bolivia':ab,ti,ca OR 'Cameroon':ab,ti,ca OR 'Cape Verde':ab,ti,ca OR 'Congo':ab,ti,ca OR 'Cote dIvoire':ab,ti,ca OR 'ivory coast':ab,ti,ca OR 'Djibouti':ab,ti,ca OR 'Egypt':ab,ti,ca OR 'El Salvador':ab,ti,ca OR 'Georgia':ab,ti,ca OR 'Ghana':ab,ti,ca OR 'Guatemala':ab,ti,ca OR 'Guyana':ab,ti,ca OR 'Honduras':ab,ti,ca OR 'Indonesia':ab,ti,ca OR 'India':ab,ti,ca OR 'Kiribati':ab,ti,ca OR 'Kosovo':ab,ti,ca OR 'Kyrgyzstan':ab,ti,ca OR 'Kyrgyz':ab,ti,ca OR 'Laos':ab,ti,ca OR 'lao':ab,ti,ca OR 'Lesotho':ab,ti,ca OR 'Mauritania':ab,ti,ca OR 'Micronesia':ab,ti,ca OR 'Moldova':ab,ti,ca OR 'Mongolia':ab,ti,ca OR 'Morocco':ab,ti,ca OR 'Nicaragua':ab,ti,ca OR 'Nigeria':ab,ti,ca OR 'Pakistan':ab,ti,ca OR 'Papua New Guinea':ab,ti,ca OR 'Paraguay':ab,ti,ca OR 'Philippines':ab,ti,ca OR 'Samoa':ab,ti,ca OR 'Atlantic Islands':ab,ti,ca OR 'Sao Tome':ab,ti,ca OR Principe:ab,ti,ca OR 'Senegal':ab,ti,ca OR 'Melanesia':ab,ti,ca OR 'Solomon islands':ab,ti,ca OR 'Sri Lanka':ab,ti,ca OR 'Sudan':ab,ti,ca OR 'Swaziland':ab,ti,ca OR 'Syria':ab,ti,ca OR 'East Timor':ab,ti,ca OR 'Timor leste':ab,ti,ca OR 'Ukraine':ab,ti,ca OR 'Uzbekistan':ab,ti,ca OR 'Vanuatu':ab,ti,ca OR 'Vietnam':ab,ti,ca OR 'Middle East':ab,ti,ca OR 'west bank':ab,ti,ca OR 'Gaza':ab,ti,ca OR 'Yemen':ab,ti,ca OR 'Zambia':ab,ti,ca OR 'Angola':ab,ti,ca OR 'Albania':ab,ti,ca OR 'Algeria':ab,ti,ca OR 'Argentina':ab,ti,ca OR 'Samoa':ab,ti,ca OR 'Azerbaijan':ab,ti,ca OR 'Republic of Belarus':ab,ti,ca OR 'Belize':ab,ti,ca OR Bosnia:ab,ti,ca OR Herzegovina:ab,ti,ca OR 'Botswana':ab,ti,ca OR 'Brazil':ab,ti,ca OR 'Bulgaria':ab,ti,ca OR 'China':ab,ti,ca OR 'Colombia':ab,ti,ca OR 'Costa Rica':ab,ti,ca OR 'Cuba':ab,ti,ca OR 'Dominica':ab,ti,ca OR 'Dominican Republic':ab,ti,ca OR 'Ecuador':ab,ti,ca OR 'Equatorial Guinea':ab,ti,ca OR 'Fiji':ab,ti,ca OR 'Gabon':ab,ti,ca OR 'Grenada':ab,ti,ca OR 'Iran':ab,ti,ca OR 'Iraq':ab,ti,ca OR 'Jamaica':ab,ti,ca OR 'Jordan':ab,ti,ca OR 'Kazakhstan':ab,ti,ca OR 'Lebanon':ab,ti,ca OR 'Libya':ab,ti,ca OR 'Macedonia':ab,ti,ca OR 'Malaysia':ab,ti,ca OR 'Indian Ocean Islands':ab,ti,ca OR 'Maldives':ab,ti,ca OR 'Marshall Islands':ab,ti,ca OR 'Mauritius':ab,ti,ca OR 'Mexico':ab,ti,ca OR 'Montenegro':ab,ti,ca OR 'Namibia':ab,ti,ca OR 'Palau':ab,ti,ca OR 'Panama':ab,ti,ca OR 'Peru':ab,ti,ca OR 'Romania':ab,ti,ca OR 'Russia':ab,ti,ca OR 'Russian Federation':ab,ti,ca OR 'Serbia':ab,ti,ca OR 'Seychelles':ab,ti,ca OR 'South Africa':ab,ti,ca OR 'Saint Lucia':ab,ti,ca OR 'Saint Vincent and the Grenadines':ab,ti,ca OR 'Suriname':ab,ti,ca OR 'Thailand':ab,ti,ca OR 'Tonga':ab,ti,ca OR 'Tunisia':ab,ti,ca OR 'Turkey':ab,ti,ca OR 'Turkmenistan':ab,ti,ca OR 'Tuvalu':ab,ti,ca OR 'Venezuela':ab,ti,ca OR 'low resource':ab,ti OR 'under resourced':ab,ti OR 'resource poor':ab,ti OR 'under developed':ab,ti OR 'underdeveloped':ab,ti OR 'developing country':ab,ti OR 'developing countries':ab,ti OR 'developing world':ab,ti OR 'third world':ab,ti OR lmic:ab,ti OR (low:ab,ti AND middle:ab,ti AND income:ab,ti) | 4112413 |
| #4 | #1 AND #2 AND #3 | 8730 |
| #5 | 'randomized controlled trial'/exp OR 'crossover procedure'/exp OR 'double blind procedure'/exp OR 'single blind procedure'/exp OR random*:ab,ti OR factorial*:ab,ti OR crossover*:ab,ti OR (cross NEAR/1 over*):ab,ti OR placebo*:ab,ti OR (doubl* NEAR/1 blind*):ab,ti OR (singl* NEAR/1 blind*):ab,ti OR assign*:ab,ti OR allocat*:ab,ti OR volunteer*:ab,ti OR trial:ab,ti OR groups:ab,ti OR Nonrandom:ab,ti OR non-random:ab,ti OR nonrandomized:ab,ti OR non-randomized:ab,ti OR nonrandomized:ab,ti OR non-randomised:ab,ti OR quasi-experiment*:ab,ti OR quasiexperiment*:ab,ti OR quasirandom*:ab,ti OR quasi-random*:ab,ti OR quasi-control*:ab,ti OR quasicontrol*:ab,ti OR (controlled:ab,ti AND (trial:ab,ti OR study:ab,ti)) OR 'clinical study'/exp OR ‘clinical trial’:ti,ab OR ‘clinical trials’:ti,ab OR 'controlled study'/exp OR 'evaluation'/exp OR ‘evaluation study’:ab,ti OR ‘evaluation studies’:ab,ti OR ‘intervention study’:ab,ti OR ‘intervention studies’:ab,ti OR ‘case control’:ab,ti OR 'cohort analysis'/exp OR cohort:ab,ti OR longitudinal*:ab,ti OR prospective:ab,ti OR prospectively:ab,ti OR retrospective:ab,ti OR 'follow up'/exp OR ‘follow up’:ab,ti OR 'comparative effectiveness'/exp OR 'comparative study'/exp OR ‘comparative study’:ab,ti OR ‘comparative studies’:ab,ti OR 'evidence based medicine'/exp OR ‘systematic review’:ab,ti OR ‘meta-analysis’:ab,ti OR ‘meta-analyses’:ab,ti NOT ('case report'/exp OR 'case study'/exp OR 'editorial'/exp OR 'letter'/exp OR 'note'/exp OR [conference abstract]/lim) | 10446677 |
| #6 | #4 AND #5 | 4343 |
| #7 | #6 AND [embase]/lim NOT [medline]/lim | 1363 |

PsycINFO

| Set | Terms | Results |
| --- | --- | --- |
| #1 | DE "Alcohol Drinking Patterns" OR DE "Alcohol Abuse" OR DE "Alcohol Intoxication" OR DE "Social Drinking" OR DE "Alcoholism" OR DE "Alcoholic Psychosis" OR DE "Binge Drinking" OR DE "Underage Drinking" OR DE "Alcoholic Beverages" OR DE "Beer" OR DE "Liquor" OR DE "Wine" OR TI (alcoholism OR ((heavy OR hazardous OR excessive OR problem OR binge OR controlled OR risky OR "at risk" OR use) AND alcohol AND drink*)) OR AB (alcoholism OR ((heavy OR hazardous OR excessive OR problem OR binge OR controlled OR risky OR "at risk" OR use) AND alcohol AND drink*)) | 76134 |
|  | DE "Psychotherapy" OR DE "Adlerian Psychotherapy" OR DE "Adolescent Psychotherapy" OR DE "Affirmative Therapy" OR DE "Analytical Psychotherapy" OR DE "Autogenic Training" OR DE "Behavior Therapy" OR DE "Brief Psychotherapy" OR DE "Brief Relational Therapy" OR DE "Child Psychotherapy" OR DE "Client Centered Therapy" OR DE "Cognitive Behavior Therapy" OR DE "Conversion Therapy" OR DE "Eclectic Psychotherapy" OR DE "Emotion Focused Therapy" OR DE "Existential Therapy" OR DE "Experiential Psychotherapy" OR DE "Expressive Psychotherapy" OR DE "Eye Movement Desensitization Therapy" OR DE "Feminist Therapy" OR DE "Geriatric Psychotherapy" OR DE "Gestalt Therapy" OR DE "Group Psychotherapy" OR DE "Guided Imagery" OR DE "Humanistic Psychotherapy" OR DE "Hypnotherapy" OR DE "Individual Psychotherapy" OR DE "Insight Therapy" OR DE "Integrative Psychotherapy" OR DE "Interpersonal Psychotherapy" OR DE "Logotherapy" OR DE "Narrative Therapy" OR DE "Network Therapy" OR DE "Persuasion Therapy" OR DE "Primal Therapy" OR DE "Psychoanalysis" OR DE "Psychodrama" OR DE "Psychodynamic Psychotherapy" OR DE "Psychotherapeutic Counseling" OR DE "Rational Emotive Behavior Therapy" OR DE "Reality Therapy" OR DE "Relationship Therapy" OR DE "Solution Focused Therapy" OR DE "Supportive Psychotherapy" OR DE "Transactional Analysis" OR DE "Counseling" OR DE "Community Counseling" OR DE "Cross Cultural Counseling" OR DE "Educational Counseling" OR DE "Gerontological Counseling" OR DE "Group Counseling" OR DE "Marriage Counseling" OR DE "Microcounseling" OR DE "Multicultural Counseling" OR DE "Occupational Guidance" OR DE "Pastoral Counseling" OR DE "Peer Counseling" OR DE "Premarital Counseling" OR DE "Psychotherapeutic Counseling" OR DE "Rehabilitation Counseling" OR DE "School Counseling" OR DE "Behavior Modification" OR DE "Behavior Therapy" OR DE "Biofeedback Training" OR DE "Contingency Management" OR DE "Fading (Conditioning)" OR DE "Omission Training" OR DE "Overcorrection" OR DE "Self-Management" OR DE "Time Out" OR DE "Cognitive Techniques" OR DE "Cognitive Restructuring" OR DE "Cognitive Therapy" OR DE "Self-Instructional Training" OR DE "Computer Assisted Therapy" OR DE "Multimodal Treatment Approach" OR DE "Social Casework" OR DE "Social Group Work" OR DE "Health Education" OR DE "Drug Education" OR DE "Family Therapy" OR DE "Conjoint Therapy" OR DE "Strategic Family Therapy" OR DE "Structural Family Therapy" OR DE "Motivational Interviewing" OR DE "Advertising" OR DE "Television Advertising" OR DE "Mass Media" OR DE "News Media" OR DE "Printed Communications Media" OR DE "Radio" OR DE "Television" OR DE "Government Policy Making" OR DE "Laws" OR DE "Drug Laws" OR DE "Health Care Policy" OR DE "Government Programs" OR DE "Mobile Devices" OR DE "Cellular Phones" OR DE "Text Messaging" OR DE "Telemedicine" OR DE "Community Mental Health Services" OR DE "Community Counseling" OR DE "Crisis Intervention Services" OR DE "Hot Line Services" OR DE "Community Psychiatry" OR DE "Community Psychology" OR DE "Community Mental Health Training" OR TI ("motivational interviewing" OR psychotherapy OR counseling OR "cognitive therapy" OR "behavior therapy" OR "behaviour therapy" OR "behavioural therapy" OR "behavioral therapy" OR "mobile applications" OR "mobile application" OR cellphone OR cellphones OR ((mobile OR cellular OR cell) AND (phone OR phones OR device OR devices OR app OR apps)) OR mobiles OR smartphone OR smartphones OR telephone OR telephones OR phone OR phones OR e-health OR ehealth OR m-health OR mhealth OR telehealth OR telemedicine OR video-conference OR videoconference OR video-conferencing OR videoconferencing OR "automated telephone" OR IVR OR "interactive voice response" OR sms OR skype OR (text AND (message OR messages)) OR texts OR mms OR blackberry OR ipad OR ipads OR android OR laptop OR laptops OR ((tablet OR tablets) AND (computer OR computers OR device OR devices)) OR ((family OR community OR school* OR psychosocial OR education OR advertis* OR billboard* OR broadcast* OR radio OR telecommunications OR media OR policy) AND (program* OR intervention*)) ) OR AB ("motivational interviewing" OR psychotherapy OR counseling OR "cognitive therapy" OR "behavior therapy" OR "behaviour therapy" OR "behavioural therapy" "behavioral therapy" OR "mobile applications" OR "mobile application" OR cellphone OR cellphones OR ((mobile OR cellular OR cell) AND (phone OR phones OR device OR devices OR app OR apps)) OR mobiles OR smartphone OR smartphones OR telephone OR telephones OR phone OR phones OR e-health OR ehealth OR m-health OR mhealth OR telehealth OR telemedicine OR video-conference OR videoconference OR video-conferencing OR videoconferencing OR "automated telephone" OR IVR OR "interactive voice response" OR sms OR skype OR (text AND (message OR messages)) OR texts OR mms OR blackberry OR ipad OR ipads OR android OR laptop OR laptops OR ((tablet OR tablets) AND (computer OR computers OR device OR devices)) OR ((family OR community OR school* OR psychosocial OR education OR advertis* OR billboard* OR broadcast* OR radio OR telecommunications OR media OR policy) AND (program* OR intervention*)) ) | 747457 |
| #3 | DE "Developing Countries" OR TI ( 'Afghanistan' OR 'Bangladesh' OR 'Benin' OR 'Burkina Faso' OR 'Burundi' OR 'Cambodia' OR 'cabo verde' OR 'Central African Republic' OR 'Chad' OR 'Comoros' OR 'Congo' OR 'Eritrea' OR 'Ethiopia' OR 'Gambia' OR 'Guinea' OR 'Haiti' OR 'Kenya' OR 'Korea' OR 'Liberia' OR 'Madagascar' OR 'Malawi' OR 'Mali' OR 'Mozambique' OR 'Myanmar' OR 'Nepal' OR 'Niger' OR 'Rwanda' OR 'Sierra Leone' OR 'Somalia' OR 'Tajikistan' OR 'Tanzania' OR 'Togo' OR 'Uganda' OR 'Zimbabwe' OR 'Armenia' OR 'Bhutan' OR 'Bolivia' OR 'Cameroon' OR 'Cape Verde' OR 'Congo' OR 'Cote dIvoire' OR 'ivory coast' OR 'Djibouti' OR 'Egypt' OR 'El Salvador' OR 'Georgia' OR 'Ghana' OR 'Guatemala' OR 'Guyana' OR 'Honduras' OR 'Indonesia' OR 'India' OR 'Kiribati' OR 'Kosovo' OR 'Kyrgyzstan' OR 'Kyrgyz' OR 'Laos' OR 'lao' OR 'Lesotho' OR 'Mauritania' OR 'Micronesia' OR 'Moldova' OR 'Mongolia' OR 'Morocco' OR 'Nicaragua' OR 'Nigeria' OR 'Pakistan' OR 'Papua New Guinea' OR 'Paraguay' OR 'Philippines' OR 'Samoa' OR 'Atlantic Islands' OR 'Sao Tome' OR Principe OR 'Senegal' OR 'Melanesia' OR 'Solomon islands' OR 'Sri Lanka' OR 'Sudan' OR 'Swaziland' OR 'Syria' OR 'East Timor' OR 'Timor leste' OR 'Ukraine' OR 'Uzbekistan' OR 'Vanuatu' OR 'Vietnam' OR 'Middle East' OR 'west bank' OR 'Gaza' OR 'Yemen' OR 'Zambia' OR 'Angola' OR 'Albania' OR 'Algeria' OR 'Argentina' OR 'Samoa' OR 'Azerbaijan' OR 'Republic of Belarus' OR 'Belize' OR Bosnia OR Herzegovina OR 'Botswana' OR 'Brazil' OR 'Bulgaria' OR 'China' OR 'Colombia' OR 'Costa Rica' OR 'Cuba' OR 'Dominica' OR 'Dominican Republic' OR 'Ecuador' OR 'Equatorial Guinea' OR 'Fiji' OR 'Gabon' OR 'Grenada' OR 'Iran' OR 'Iraq' OR 'Jamaica' OR 'Jordan' OR 'Kazakhstan' OR 'Lebanon' OR 'Libya' OR 'Macedonia' OR 'Malaysia' OR 'Indian Ocean Islands' OR 'Maldives' OR 'Marshall Islands' OR 'Mauritius' OR 'Mexico' OR 'Montenegro' OR 'Namibia' OR 'Palau' OR 'Panama' OR 'Peru' OR 'Romania' OR 'Russia' OR 'Russian Federation' OR 'Serbia' OR 'Seychelles' OR 'South Africa' OR 'Saint Lucia' OR 'Saint Vincent and the Grenadines' OR 'Suriname' OR 'Thailand' OR 'Tonga' OR 'Tunisia' OR 'Turkey' OR 'Turkmenistan' OR 'Tuvalu' OR 'Venezuela' OR 'low resource' OR 'under resourced' OR 'resource poor' OR 'under developed' OR 'underdeveloped' OR 'developing country' OR 'developing countries' OR 'developing world' OR 'third world' OR lmic OR (low AND middle AND income) ) OR AB ( 'Afghanistan' OR 'Bangladesh' OR 'Benin' OR 'Burkina Faso' OR 'Burundi' OR 'Cambodia' OR 'cabo verde' OR 'Central African Republic' OR 'Chad' OR 'Comoros' OR 'Congo' OR 'Eritrea' OR 'Ethiopia' OR 'Gambia' OR 'Guinea' OR 'Haiti' OR 'Kenya' OR 'Korea' OR 'Liberia' OR 'Madagascar' OR 'Malawi' OR 'Mali' OR 'Mozambique' OR 'Myanmar' OR 'Nepal' OR 'Niger' OR 'Rwanda' OR 'Sierra Leone' OR 'Somalia' OR 'Tajikistan' OR 'Tanzania' OR 'Togo' OR 'Uganda' OR 'Zimbabwe' OR 'Armenia' OR 'Bhutan' OR 'Bolivia' OR 'Cameroon' OR 'Cape Verde' OR 'Congo' OR 'Cote dIvoire' OR 'ivory coast' OR 'Djibouti' OR 'Egypt' OR 'El Salvador' OR 'Georgia' OR 'Ghana' OR 'Guatemala' OR 'Guyana' OR 'Honduras' OR 'Indonesia' OR 'India' OR 'Kiribati' OR 'Kosovo' OR 'Kyrgyzstan' OR 'Kyrgyz' OR 'Laos' OR 'lao' OR 'Lesotho' OR 'Mauritania' OR 'Micronesia' OR 'Moldova' OR 'Mongolia' OR 'Morocco' OR 'Nicaragua' OR 'Nigeria' OR 'Pakistan' OR 'Papua New Guinea' OR 'Paraguay' OR 'Philippines' OR 'Samoa' OR 'Atlantic Islands' OR 'Sao Tome' OR Principe OR 'Senegal' OR 'Melanesia' OR 'Solomon islands' OR 'Sri Lanka' OR 'Sudan' OR 'Swaziland' OR 'Syria' OR 'East Timor' OR 'Timor leste' OR 'Ukraine' OR 'Uzbekistan' OR 'Vanuatu' OR 'Vietnam' OR 'Middle East' OR 'west bank' OR 'Gaza' OR 'Yemen' OR 'Zambia' OR 'Angola' OR 'Albania' OR 'Algeria' OR 'Argentina' OR 'Samoa' OR 'Azerbaijan' OR 'Republic of Belarus' OR 'Belize' OR Bosnia OR Herzegovina OR 'Botswana' OR 'Brazil' OR 'Bulgaria' OR 'China' OR 'Colombia' OR 'Costa Rica' OR 'Cuba' OR 'Dominica' OR 'Dominican Republic' OR 'Ecuador' OR 'Equatorial Guinea' OR 'Fiji' OR 'Gabon' OR 'Grenada' OR 'Iran' OR 'Iraq' OR 'Jamaica' OR 'Jordan' OR 'Kazakhstan' OR 'Lebanon' OR 'Libya' OR 'Macedonia' OR 'Malaysia' OR 'Indian Ocean Islands' OR 'Maldives' OR 'Marshall Islands' OR 'Mauritius' OR 'Mexico' OR 'Montenegro' OR 'Namibia' OR 'Palau' OR 'Panama' OR 'Peru' OR 'Romania' OR 'Russia' OR 'Russian Federation' OR 'Serbia' OR 'Seychelles' OR 'South Africa' OR 'Saint Lucia' OR 'Saint Vincent and the Grenadines' OR 'Suriname' OR 'Thailand' OR 'Tonga' OR 'Tunisia' OR 'Turkey' OR 'Turkmenistan' OR 'Tuvalu' OR 'Venezuela' OR 'low resource' OR 'under resourced' OR 'resource poor' OR 'under developed' OR 'underdeveloped' OR 'developing country' OR 'developing countries' OR 'developing world' OR 'third world' OR lmic OR (low AND middle AND income)) | 182279 |
| #4 | S1 AND S2 AND S3 | 885 |
| #5 | ZC "longitudinal study" OR ZC "empirical study" OR ZC "followup study" OR ZC "longitudinal study" OR ZC "meta analysis" OR ZC "prospective study" OR ZC "retrospective study" OR ZC "systematic review" OR ZC "treatment outcome/clinical trial" OR DE "Clinical Trials" OR DE "Cohort Analysis" OR DE "Followup Studies" OR DE "Longitudinal Studies" OR DE "Prospective Studies" OR DE "Meta Analysis" OR TI (randomized OR randomised OR randomization OR randomisation OR randomly OR trial OR groups OR trials OR "evaluation study" OR evaluation studies OR "intervention study" OR "intervention studies" OR "case-control" OR cohort OR longitudinal OR longitudinally OR prospective OR prospectively OR retrospective OR "comparative study" OR "meta-analysis" OR "meta-analyses" OR "systematic review" OR "systematic reviews" OR groups OR Nonrandom OR non-random OR nonrandomized OR non-randomized OR nonrandomized OR non-randomised OR quasi-experiment* OR quasiexperiment* OR quasirandom* OR quasi-random* OR quasi-control* OR quasicontrol* OR (controlled AND (trial OR study))) OR AB (randomized OR randomised OR randomization OR randomisation OR randomly OR trial OR groups OR trials OR "evaluation study" OR evaluation studies OR "intervention study" OR "intervention studies" OR "case-control" OR cohort OR longitudinal OR longitudinally OR prospective OR prospectively OR retrospective OR "comparative study" OR "meta-analysis" OR "meta-analyses" OR "systematic review" OR "systematic reviews" OR groups OR Nonrandom OR non-random OR nonrandomized OR non-randomized OR nonrandomized OR non-randomised OR quasi-experiment* OR quasiexperiment* OR quasirandom* OR quasi-random* OR quasi-control* OR quasicontrol* OR (controlled AND (trial OR study))) AND (ZZ "journal article") | 2365430 |
| #6 | #4 AND #5 | 672 |

WHO Global Health Library – regional indices

| Set | Terms | Results |
| --- | --- | --- |
| #1 | ((heavy OR hazardous OR harmful OR excessive OR problem OR binge OR controlled OR risky OR "at risk" OR "at-risk" OR use) AND alcohol AND drink*) OR alcoholism |  |
| #2 | Intervention OR interventions OR "motivational interviewing" OR psychotherapy OR counseling OR "cognitive therapy" OR "behavior therapy" OR "behaviour therapy" OR "behavioural therapy" OR "behavioral therapy" OR advertis* OR billboard* OR broadcast* OR radio OR telecommunications OR media OR policy OR "mobile applications" OR "mobile application" OR cellphone OR cellphones OR ((mobile OR cellular OR cell) AND (phone OR phones OR device OR devices OR app OR apps)) OR mobiles OR smartphone OR smartphones OR telephone OR telephones OR phone OR phones OR e-health OR ehealth OR m-health OR mhealth OR telehealth OR telemedicine OR video-conference OR videoconference OR video-conferencing OR videoconferencing OR "automated telephone" OR IVR OR "interactive voice response" OR sms OR skype OR (text AND (message OR messages)) OR texts OR mms OR blackberry OR ipad OR ipads OR android OR laptop OR laptops OR ((tablet OR tablets) AND (computer OR computers OR device OR devices)) OR ((family OR community OR school* OR psychosocial OR education) AND (program* OR intervention*)) |  |
| #3 | randomized OR randomised OR randomization OR randomisation OR randomly OR trial OR groups OR Nonrandom OR non-random OR nonrandomized OR non-randomized OR nonrandomized OR non-randomised OR quasi-experiment* OR quasiexperiment* OR quasirandom* OR quasi-random* OR quasi-control* OR quasicontrol* OR "evaluation study" OR evaluation studies OR "intervention study" OR "intervention studies" OR "case-control" OR cohort OR longitudinal OR longitudinally OR prospective OR prospectively OR retrospective OR "follow up" OR "comparative study" OR systematic OR "meta-analysis" OR "meta-analyses" |  |
| #4 | #1 AND #2 AND #3, limited to LILACS, WPRIM, WHOLIS, IMSEAR, IMEMR | 476 |
