# Supplementary figures and images for: Patient-level interventions to reduce alcohol-related harms in low- and middle-income countries: A systematic review and meta-summary
Source: PLoS Med. 2022 Apr 12;19(4):e1003961. doi: 10.1371/journal.pmed.1003961 (PMC9004752; doi:10.1371/journal.pmed.1003961)

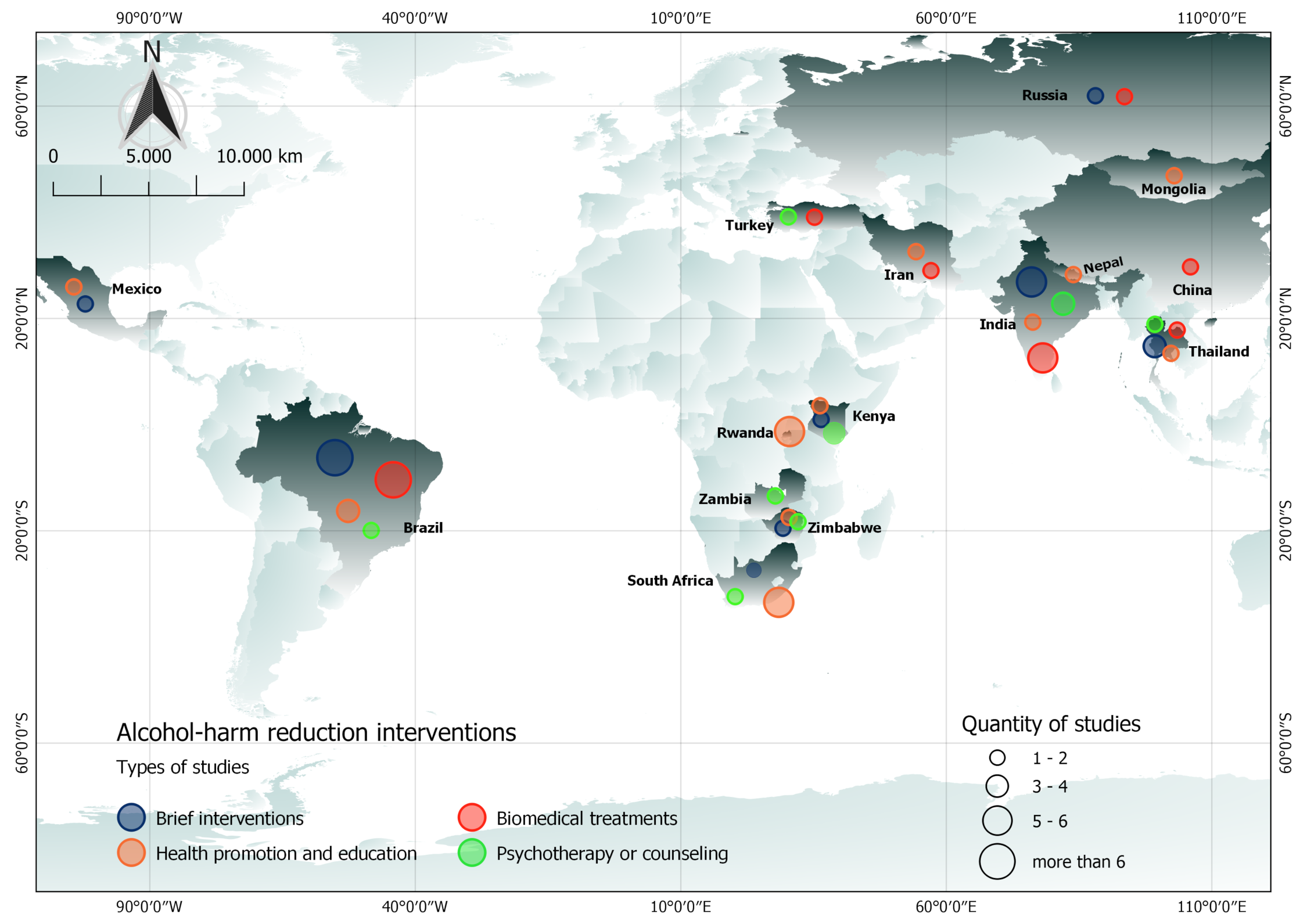

Supplement: S2 Fig — Source: Global Administrative Areas (2022). University of California, Berkely. Available online: http://www.gadm.org [11/03/2022]; https://geodata.ucdavis.edu/gadm/gadm4.0/gadm404-shp.zip. (TIF) [file pmed.1003961.s002.tif]
