# Supplementary material for: Patient-level interventions to reduce alcohol-related harms in low- and middle-income countries: A systematic review and meta-summary
Source: PLoS Med. 2022 Apr 12;19(4):e1003961. doi: 10.1371/journal.pmed.1003961 (PMC9004752; doi:10.1371/journal.pmed.1003961)
Supplement: Attachment — Submitted filename: Plos Med Rebuttal Letter.pdf [file pmed.1003961.s005.pdf]

Duke Emergency Medicine
A Division of the Department of Surgery

DUKE GLOBAL HEALTH INSTITUTE

January 7th, 2020

Dear PloS Medicine Reviewers,

Thank you for the careful review of our submission entitled "Interventions to Reduce Alcohol-Related Harm in Low- and Middle-Income Countries: a Systematic Review and Meta-Summary" (PMEDICINE-D-19-02160) for consideration at PLOS Medicine in the upcoming special issue on substance mis/use. The very helpful revisions requested required a full re-analysis of the project. As such, our systematic review has searched 4370 articles which found 101 articles fitting our inclusion and exclusion criteria, of which 62 were RCTs which evaluated a patient level intervention targeting alcohol use and alcohol related harm. Brief interventions (19 of 62 articles) was most commonly evaluated and motivational interviewing techniques were most common utilized in these studies. Both brief interventions and motivational interviewing techniques also had the most consistent positive effect. Unfortunately, our review found very high heterogeneity in intervention types, outcome measures, and follow up times limiting our ability to perform a meta-analysis or make further inferences about the most effective interventions.

Again we thank you for the opportunity to revise our manuscript. We have carefully reviewed the comments and have responded as follows:

Requests from the editors:

1. We ask you to update your search to a date in the last few months.
RESPONSE: Yes, thank you for the opportunity to update the search to the current date to ensure the most up to date review.

2. Please restructure the abstract of your paper so that there are three sections: background, methods and findings, and conclusion. The final sentence of the "methods and findings" subsection should summarize the study's main limitations.
RESPONSE: We have restructured the abstract into these three mentioned categories and included a summary sentence.

3. Please add a sentence, say, to your abstract detailing the interventions that have showed promise in randomized controlled evaluations.
RESPONSE: We have added further interpretation about the most promising types of studies and methods used to reduce alcohol related outcomes.

4. After your abstract, we will need to ask you to add a new and accessible "author summary" section in non-identical prose. You may find it helpful to consult one or two recent research papers published in PLOS Medicine to get a sense of the preferred style.
RESPONSE: We have included a new section "Author Summary" in non-identical prose with the sections "Why was this study done?," "What did the researchers do and find?," and "What do these findings mean?".

5. Throughout your text, please ensure that reference call-outs fall before punctuation, e.g. "... follow-up [42].".
RESPONSE: We have carefully reviewed throughout the text to ensure reference call-outs fall before punctuation.

6. Where you make a claim of "the first", e.g. at line 236, please add "to our knowledge" or similar.
RESPONSE: We added "to our knowledge" to that line.

7. In the reference list, please ensure that journal names are abbreviated as appropriate, e.g. "Lancet" rather than "the lancet".
RESPONSE: We have carefully reviewed the references in order to ensure the journal names are abbreviated as appropriate

8. Please complete references 50 & 78, and provide additional information for reference 14 & 21 (including URLs if available, along with accessed dates).
RESPONSE: We have completed these references as requested.

9. In figure 1, two boxes contain "(n =)" which we ask you to complete as appropriate.
RESPONSE: We have updated figure 1 as requested.

10. Please adapt the attached PRISMA checklist so that individual items are referred to by section (e.g., "Methods") and paragraph number rather than by line or page numbers, as the latter generally change in the event of publication.
RESPONSE: We have updated the PRISMA checklist as requested.

<u>Comments from the reviewers:</u>

***** Reviewer #1:**

I confine my remarks to statistical aspects of this paper. The general approach is fine, but I have some issues to resolve before I can recommend publication.

1. Line 45 - No meta-analysis appears to have been done. I think this is the right choice, but it makes this line incorrect.
RESPONSE: Thank you for pointing this out. You are correct, we only conducted a meta-summary and the introduction, methods and abstract were updated to state this.

2. Line 62 - I don't think "standardize" is the right word here. I am not sure what the authors mean. Maybe it is the interventions that need to be standardized?
RESPONSE: Thank you, we think that the 'standardized' word you are speaking of was in the abstract. We agree this was confusing and this sentence was removed.

3. Figure 1 - there are two blanks (e.g. records after duplicates removed). Also, the box for articles excluded that were not RCT should come out of "full text articles assessed". Lastly, I think the bottom box should either get N = 0 or be dropped.
RESPONSE: Thank you for pointing out this oversight. These numbers were included in the figure.

***** Reviewer #2:**

1) Sorry, but the title and search terms would suggest that all interventions were taken into consideration. This would include the "best buys" (taxation increases, ban on marketing, limitations of availability). Since these interventions have been designated by WHO and others as the most appropriate also in LMIC, I find it curious that they were left out without good reasoning.

RESPONSE: Thank you for pointing this out, we are sorry for this oversight. This intervention was performed as the first step to the creation of a patient level intervention in a low income setting. As such, we wanted to focus on patient centered alcohol harm reduction interventions. We have changed the title, the introduction, methods and results in order to better state and justify this. While some of the health promotion and education interventions are evaluated on the community level, they were administered at the patient level and as such they were included in this systematic review.

2) Where was it stated that the final studies had to be RCTs? This has to be made more explicit.

RESPONSE: Since we didn't know the extent or quality of the literature in LMICs, we were more inclusive of study type. Therefore, we included all study types in our search strategy. After our initial search was identified numerous articles, we then were able to report preliminary data on all the studies to describe the literature in general. Next we looked specifically at the randomized controlled trials to understand which methods had the best efficacy. This was further explained in the methods under eligibility and study selection

3) Title and descriptions have to be changed to correspond to the limitations of the actual design.

RESPONSE: Yes, thank you for this suggestion. We have updated the title and the methods to better describe the methods and the limitations of our design.

4) Reference list at times sloppy without page numbers. Also, of course, the searches should have been wider with different data bases, if in the end only individual-based interventions were selected.

RESPONSE: Thank you for this suggestion. We have updated our references to include page numbers as needed. Similarly, based on our updated objectives to identify patient-level interventions we focused our database on those which would best give us those evaluations. We included 7 databases and one specifically for global health literature (WHO Global Health LIterature) in order to ensure we captured all available articles which far exceeds PRISMA recommendations for number of databases for systematic reviews.

5) Minor point: status of WB classification should be mid-term of the interventions and not date of publication.

RESPONSE: Thank you for this point, and you are correct, optimally the country's World Bank status should be determined at the midpoint of the intervention. We searched specifically for high income status at the time of the pull; we acknowledge that this would limit those who have since become high income or those who have ceased to be countries as recognized by the world bank. We included this in the limitations and for future studies.

6) Final point: while the authors are technically right to indicate the different measurement of outcome, the variance for ALL measures is de facto determined by QF by > 60% -> this means Z-standardization and meta-analysis would be possible.

RESPONSE: We appreciate the comment and acknowledge that indeed we could analyize the outcomes as suggested by the reviewer. We chose not to go forward with the meta-analysis due to the

varied methodologies (population, intervention, follow up times and so on) and outcomes used, focusing on a summary approach to the variability in the literature.

<u>* Reviewer #3:</u>**

This paper represents a carefully done systematic review of mostly clinical studies for alcohol treatment. The introduction makes a compelling case for the importance of reviewing what is available of effective individual-level interventions, given the importance of reducing hazardous drinking. This could be a great compendium to be used as a resource. There are however some important areas that need to be addressed to make a significant contribution.

1. First, the abstract and introduction should not have categorized the study as a meta-analysis but rather as a systematic review.
   RESPONSE: Thank you for pointing this out, we have made this changes as suggested.

2. Second, the title should clarify that this systematic review is not of all types of interventions as stated in the introduction ("review and describe the current published literature on alcohol interventions in LMICs") but rather limited to a certain group of interventions. The Discussion section should clarify that many other types of interventions are excluded (i.e. policy, social marketing) from the review, including population-based interventions.
   RESPONSE: Thank you for pointing out this oversight. The title and the text (introduction, methods, results and discussion) were all changed in order to better reflect that we did a systematic review specifically of patient level interventions.

3. Third, the criteria and words used for the search do not explicitly say that they had to be randomized trials, or the wording to only include brief interventions, health education, medication, psychotherapy, or brain stimulation studies. How did the authors decide on these types of interventions? Nor do the authors state why other types of studies were excluded.
   RESPONSE: Since we didn't know the extent or quality of the literature in LMICs, we were more inclusive of study type. Therefore, we included all study types in our search strategy. After our initial search was identified numerous articles, we then were able to report preliminary data on all the studies to describe the literature in general. Next we looked specifically at the randomized controlled trials to understand which methods had the best efficacy. This was further explained in the methods under eligibility and study selection

4. The abstract says all languages were included but state that "we found no manuscripts in other languages." Could it be due to the data sources included? Given the large body of studies in South America, this seem as a surprise.
   RESPONSE: We had no language limitations on our database searches. Still we found no non-english language manuscripts. We have repeated the search and still found no non-english language manuscripts to add to our review.

5. Fourth, Figure 1 needs to be revised because there is missing information (n=?).
   RESPONSE: This has been revised.

6. Other concerns deal with the constant shift describing settings (where the interventions were done), type of population served by the interventions, without acknowledging whether the content of the alcohol intervention component of the interventions differed. At other times, when describing the studies, more detail was provided of the content but again there was a constant

shift in what of the interventions was being described (i.e. setting, population, content, type of evaluation, format of the intervention, results of the intervention, country where the intervention was conducted, time of the assessment). This made following the results of the systematic review extremely cumbersome. A more consistent approach to describing the studies is necessary to help guide the reader.

RESPONSE: Thank you for this very appropriate feedback. We have since rewritten the whole methods, results and most of the discussion to have more focused reporting, and similar writing.

7. The idea that a nurse can deliver the intervention and "even" be effective seems to undermine the value of nurses. Can the authors justify?

RESPONSE: Thank you and we agree with your comment. We have removed this inappropriate statement and reframed this section of the results given the new data interpretation and manuscripts found on a complete update of the search.

8. The writing in some sections also seems choppy (lines 203-205, 218 and others) and sometimes unclear. For example, what do the authors mean by: "psychotherapy or counseling most frequently utilized a blend of theoretical bases"; "limited standardization for outcomes across studies"; "wide variability in the metric of improvement chosen"; "standardization of outcome measures as well as designing interventions"; or "augmentation with additional types of treatment"?

RESPONSE: Thank you for these comments. Our language was reviewed in order to ensure more clear and concise verbiage.

9. Also unclear was why in some sections the authors of the interventions are mentioned but not in other sections, seemingly as if different sections of the paper were written by different authors.

RESPONSE: Thank you for this comment. We have rewritten the whole results and discussion sections with the new database pull and updated results and have ensured that the sections are coherent and have a logical flow.

10. The Discussion section should specify why the authors focused on alcohol harm reduction interventions, which is only stated at the end of the synthesis but not mentioned as a term in the search for topics.

RESPONSE: Thank you for this comment. This information has been added.
