# Supplementary material for: Patient-level interventions to reduce alcohol-related harms in low- and middle-income countries: A systematic review and meta-summary
Source: PLoS Med. 2022 Apr 12;19(4):e1003961. doi: 10.1371/journal.pmed.1003961 (PMC9004752; doi:10.1371/journal.pmed.1003961)
Supplement: Attachment — Submitted filename: Reviewer 3 Comments D-19-02160.docx [file pmed.1003961.s004.docx]

Reviewer 3 Comments: Minor Revision

This paper represents a carefully done systematic review of mostly clinical studies for alcohol treatment. The introduction makes a compelling case for the importance of reviewing what is available of effective individual-level interventions, given the importance of reducing hazardous drinking. This could be a great compendium to be used as a resource. There are however some important areas that need to be addressed to make a significant contribution.

First, the abstract and introduction should not have categorized the study as a meta-analysis but rather as a systematic review. Second, the title should clarify that this systematic review is not of all types of interventions as stated in the introduction (“review and describe the current published literature on alcohol interventions in LMICs”) but rather limited to a certain group of interventions. The Discussion section should clarify that many other types of interventions are excluded (i.e. policy, social marketing) from the review, including population-based interventions. Third, the criteria and words used for the search do not explicitly say that they had to be randomized trials, or the wording to only include brief interventions, health education, medication, psychotherapy, or brain stimulation studies. How did the authors decide on these types of interventions? Nor do the authors state why other types of studies were excluded. The abstract says all languages were included but state that “we found no manuscripts in other languages.” Could it be due to the data sources included? Given the large body of studies in South America, this seem as a surprise. Fourth, Figure 1 needs to be revised because there is missing information (n=?).

Other concerns deal with the constant shift describing settings (where the interventions were done), type of population served by the interventions, without acknowledging whether the content of the alcohol intervention component of the interventions differed. At other times, when describing the studies, more detail was provided of the content but again there was a constant shift in what of the interventions was being described (i.e. setting, population, content, type of evaluation, format of the intervention, results of the intervention, country where the intervention was conducted, time of the assessment). This made following the results of the systematic review extremely cumbersome. A more consistent approach to describing the studies is necessary to help guide the reader.

The idea that a nurse can deliver the intervention and “even” be effective seems to undermine the value of nurses. Can the authors justify? The writing in some sections also seems choppy (lines 203-205, 218 and others) and sometimes unclear. For example, what do the authors mean by: “psychotherapy or counseling most frequently utilized a blend of theoretical bases”; “limited standardization for outcomes across studies”; “wide variability in the metric of improvement chosen”; “standardization of outcome measures as well as designing interventions”; or “augmentation with additional types of treatment”? Also unclear was why in some sections the authors of the interventions are mentioned but not in other sections, seemingly as if different sections of the paper were written by different authors.

The Discussion section should specify why the authors focused on alcohol harm reduction interventions, which is only stated at the end of the synthesis but not mentioned as a term in the search for topics.
