# Supplementary material for: Patient-level interventions to reduce alcohol-related harms in low- and middle-income countries: A systematic review and meta-summary
Source: PLoS Med. 2022 Apr 12;19(4):e1003961. doi: 10.1371/journal.pmed.1003961 (PMC9004752; doi:10.1371/journal.pmed.1003961)
Supplement: Attachment — Submitted filename: SecondPlos Med Rebuttal Letter F.docx [file pmed.1003961.s006.docx]

February 9, 2021

Dear PloS Medicine Reviewers,

Thank you for the careful review of our submission entitled "Interventions to Reduce Alcohol-Related Harm in Low- and Middle-Income Countries: a Systematic Review and Meta-Summary" (PMEDICINE-D-19-02160)

The very helpful revisions requested required a full re-analysis of the project. As such, our systematic review has searched 5036 articles which found 117 articles fitting our inclusion and exclusion criteria, of which 75 were RCTs which evaluated a patient level intervention targeting alcohol use and alcohol related harm. As we had to repull manuscripts for the last year and reanalyze data, we are requesting permission to add two paired authors (CY and AP) to the author list who were responsible for conducting both data analysis and editing of the manuscript to allow for these revisions to take place. We have obtained permission by email from our editor to do so as requested.

As such, our new authors should be:

**Authors**

*Catherine A. Staton, MD MScGH^1,2^, Joao Ricardo Nickenig Vissoci, PhD^1,2^ , Deena El-Gabri^2^, Konyinsope Adewumi^2^ ,Tessa Concepcion^2^, Shannon A. Elliott^2^, Daniel R. Evans^2^, Sophie W. Galson^1, 2^, Charles T. Pate^2^ , Lindy M. Reynolds^2^, Nadine A. Sanchez^2^, Alexandra E. Sutton, PhD^2,3^, Charlotte Yuan^2^ Alena Pauley^2^, Luciano Andrade, RN PhD^4^, Megan Von Isenberg^5^, Jinny J. Ye, MD^1^, Charles J. Gerardo, MD, MHS^1,2^

**emails**

*Catherine Staton, MD MScGH^1,2,^	catherine.staton@duke.edu

Joao Ricardo Vissoci, PhD^1,2,^	jnv4@duke.edu

Deena El-Gabri^2^	deenaelgabri@gmail.com

Konyinsope Adewumi^2^	khadewumi@gmail.com

Tessa Concepcion^2^	tessaconcepcion@outlook.com

Shannon A. Elliott^2^	shannon.elliott@gmail.com

Daniel R. Evans^2^	evans.danielrowland@gmail.com

Sophie W. Galson^1, 2^	swgalson@gmail.com

Charles T. Pate^2^	charlespate12@gmail.com

Lindy M. Reynolds^2^	lindyreynolds7@gmail.com

Nadine A. Sanchez^2^	nadineamanda@ucla.edu

Alexandra E. Sutton, PhD^2,3^	lexasutton@gmail.com

Charlotte Yuan^2^	yunyuan98@gmail.com

Alena Pauley^2^	alena.pauley@gmail.com

Luciano Andrade, RN PhD^4^	landrade@uem.br

Megan Von Isenberg^5^	Megan.vonisenberg@duke.edu

Jinny J. Ye, MD^1^	jinny.ye@duke.edu

Charles J. Gerardo, MD, MHS^1,2^	charles.gerardo@duke.edu

1 Duke Division of Emergency Medicine, Department of Surgery, Duke University Medical Center, Duke University, Durham, NC

2 Duke Global Health Institute, Duke University, Durham, NC

3 Nicholas School of the Environment, Duke University, Durham, NC

4 Department of Medicine, State University of Maringa, Maringa, Parana State, Brazil

5 Duke School of Medical Center Library Services & Archives, Duke University, Durham, NC

Again we thank you for the opportunity to revise our manuscript. We have carefully reviewed the comments and have responded as follows:

**Editor Comments:**

**-clean version of the paper as the main article file;**

Response: We will update a clean version of the paper as the main article file

**-a version with changes marked should be uploaded as a marked up manuscript.**

Response: We have uploaded a version with changes marked as a marked up manuscript

**-upload any figures associated with your paper as individual TIF or EPS files with 300dpi resolution at resubmission;**

Response: We have uploaded images as TIF files with 300dpi

**Please finalize the arrangements for data deposition.**

Response: Yes, we have arranged for a data deposition.

**Please update the search to a date in the last 3 months.**

Response: The search was updated to December 1, 2020.

**Please quote the date of the search in the abstract. Also, please quote the proportions of studies carried out in the different country categories - low-income countries, etc.**

Response: This was added to the abstract

**We suggest adding "people in" at line 37.**

Response: This was added where requested.

**Please revisit the phrasing at line 61, where the second "that" should perhaps be removed.**

Response: Rephrasing was performed and the first ‘that’ was removed for clarity.

**It seems that motivational interviewing interventions fall into both the "brief interventions" and "psychotherapy" categories. Please detail the key differences underlying these categorizations.**

Response: You are very correct. We further delineated these two categories in the methods as such: Using the WHO and NIAAA descriptions of brief interventions, we defined brief interventions as any intervention ranging from advice to counselling, lasting less than 1 hour per session {Henry-Edwards, 2003 #3827} up to 4 sessions {NIAAA, 2005 #3831} independent of how the original study defined brief intervention. Interventions including a one-on-one interaction with a counselor that lasted longer than a brief intervention or that included a psychotherapeutic component were defined as Psychotherapy and counseling. Motivational interviewing techniques could be included in either the ‘brief intervention’ or the ‘psychotherapy and counseling’ depending on how long and over how many sessions the intervention took place.

**At line 128, please refer to the attached checklist (e.g., "See S1_PRISMA_Checklist") and rename the attachment to match.**

Response: This was added where requested and was saved by the referenced title.

**Throughout the text, please remove spaces from within the reference call-outs, e.g., " control arms equally [26,71].".**

Response: This was removed throughout as requested.

**Please remove the information on financial disclosures from the end of the main text. In the event of publication, this information will appear in the article metadata via information provided in the submission form.**

Response: This was removed where requested.

**In the reference list, please use journal name abbreviations, e.g., "PLoS Med.", consistently.**

Response: All references were updated as requested.

**Please spell out fully the institutional author name for reference 3 and any other relevant references.**

Response: All references were updated as requested.

**Please ensure that all references contain full access details, e.g., reference 9.**

Response: All references were updated as requested especially #9.

**We did not find supplementary table 1 with the paper - please ensure that this is provided with your resubmission.**

Response: We are very sorry. There should be two supplements. S1 is the search strategy and S2 is the PRISMA_Checklist. They will both be uploaded with this resubmission.

***** Reviewer #1: The authors have addressed my concerns and I now recommend publication**

Response: Thank you so much for this recommendation.

***** Reviewer #2:**

**While the review improved substantially, I still see problems with this revised version (Referring to the version marked):**

**1) The new title is: "Patient-level Interventions to Reduce Alcohol-Related Harm ...**

**Sorry but brief interventions by definition are not directed at patients -> see WHO manual on brief interventions.**

Response: Patient Level interventions is stating it this way in order to separate it from health policy level interventions to reduce alcohol related harms. We are stating this as ‘patient-level’ rather than patient directed specifically due to this challenge.

**2) The interventions are now dichotomized at 'brief interventions' and biological treatment. Most treatment interventions are not biological but talk therapy.**

Response: Thank you for this comment. We have categorized the interventions into ‘brief interventions’ ‘psychotherapy and counseling’ ‘health promotion’ and ‘biological’. We agree that most treatments are NOT ‘biological’ but this categorization represents the diversity of the interventions that we found from our search.

**3) This reviewer is still concerned with the lack of a meta-analysis, which would be possible.**

Response: As further delineated in this version of the manuscript, we do not believe that a meta-analysis is possible because of the range of outcome measures and different time points for each of these 117 studies. As such, we are not performing a meta-analysis but a meta-summary for these reasons.

**4) The references continue to be sloppy and old.**

Response: Thank you for this feedback. We have gone through each and every one of the references to make sure they are the most up to date and appropriate citations.

***** Reviewer #3:**

**This papers has been drastically improved and can be informative of the literature available on patient-level alcohol-related interventions in low and middle income countries. I do have a series of comments that require final attention.**

**Please pass speller to ensure that there are no typographical errors such as "analyses".**

Response: Thank you for this feedback. We used spell check and had an editor edit this version prior to resubmission.

**The abstract and highlights of the study indicate that the most consistently positive results were brief interventions. "Brief interventions" does not qualify what types of interventions or define it. There is a plethora of brief interventions so the reader needs to have a more concrete picture to what types of interventions. Please use the definition in lines 198-200 to specify what you mean from the beginning of the manuscript.**

Response: You are very correct, brief interventions are a broad range of interventions. We have reiterated this definition in the introduction to ensure readers understand our use of the term and categorization.

**Given the absence of conclusive evidence of effective alcohol interventions in LMIC, is it adequate to recommend "further evaluations should focus on the most effective implementation strategies in low- and middle-income settings?" Or should it be on testing the comparative effectiveness of these brief interventions in different LMIC contexts to determine what works for whom and under what conditions? This is particularly important since the authors state that most studies are "limited to middle-income settings" and that "lack of uniformity compromised our ability to discern the effectiveness of interventions."**

Response: This is a great point. Honestly, both/all are needed. We have changed this point to state that comparative effectiveness and implementation strategies including target population delineation is needed.

**There is some confusion in stating that meta-analyses and systematic reviews were excluded, and then saying that there were no systematic reviews or meta-analysis in LMIC. Which of the two is it? It cannot be both.**

Response: Thank you for this observation. We have corrected our introduction to highlight that we found no systematic review in our literature search, but yes, during our systematic review portion of the project we omitted systematic reviews and included only primary literature.

**The statement in line 142 "Studies which had the same population represented had the most recent data included in the review" is not clear. Please clarify if what you mean is that if two studies used the same data, the most recent study was selected for representation.**

Response: Sorry for this confusion. This sentence was changed as suggested. “If two studies used the same data, then the most recent data was included in the review.

**Please clarify what you mean by "No studies were excluded for language" bit then authors state "the datasets we have searched are not extensive enough to include non-English language articles."**

Response: Thank you for this comment. We have further explained this limitation as such: “First, our search strategy did not exclude studies due to language and yet we found no manuscripts in other languages. Thus, either there is no non-English language literature on this topic or the datasets we searched have limited non-English language articles.”

**In lines 304-305, it is unclear if the arms are intervention arms and the comparison is across different treatments or across a treatment and a control arm. Please specify.**

Response: We are sorry for this confusing sentence. We have edited this to be more clear stating: “A few of the studies in this subgroup had null effects or found no difference between the intervention and control arms.”

**Finally, some sentences need to be rephrase for clarity: "Given infrastructure in low-income settings has even more limited mental health and substance abuse facilities and professionals, and greater associated stigma; implementation of this kind of intervention is still potentially unfeasible."**

Response: Thank you for pointing this out. We have done a complete review of the manuscript and have had an editor also read through it.

**Overall, this manuscript has great potential to be of interest to readers of the journal but requires further attention.**

Thank you for the opportunity to revise our manuscript.

Catherine A. Staton, MD MScGH

Associate Professor of Emergency Medicine and Global Health

Director, Global Emergency Medicine Innovation and Implementation (GEMINI) Research Lab

EM Director, Hubert Yeargan Global Health Residency and Fellowship Pathway

Division of Emergency Medicine, Department of Surgery

Duke University School of Medicine

Duke Global Health Institute
