# Supplementary material for: Patient-level interventions to reduce alcohol-related harms in low- and middle-income countries: A systematic review and meta-summary
Source: PLoS Med. 2022 Apr 12;19(4):e1003961. doi: 10.1371/journal.pmed.1003961 (PMC9004752; doi:10.1371/journal.pmed.1003961)
Supplement: Attachment — Submitted filename: 3rd rebuttle letter.docx [file pmed.1003961.s007.docx]

February 28, 2022

Dear PloS Medicine Reviewers,

Thank you for the provisional acceptance of our submission entitled "Interventions to Reduce Alcohol-Related Harm in Low- and Middle-Income Countries: a Systematic Review and Meta-Summary" (PMEDICINE-D-19-02160)

We have carefully addressed the remaining editorial and production issues, We have responded as follows:

1. Please ensure that the paper adheres to the PLOS Data Availability Policy (see <https://urldefense.com/v3/__http://journals.plos.org/plosmedicine/s/data-availability__;!!OToaGQ!_4SUmamBRBv_yABvRjM3gAIJVkaQCh4uEkKx0KHg7udvJxvaLtcCIdAyS8Jl1Fk9X9Zpyg$> ), which requires that all data underlying the study's findings be provided in a repository or as Supporting Information. For data residing with a third party, authors are required to provide instructions with contact information for obtaining the data. PLOS journals do not allow statements supported by "data not shown" or "unpublished results." For such statements, authors must provide supporting data or cite public sources that include it.

   **R: We have revised and confirm the paper adheres to the PLOS Data Availability Policy**
2. Please review your reference list to ensure that it is complete and correct. If you have cited papers that have been retracted, please include the rationale for doing so in the manuscript text, or remove these references and replace them with relevant current references. Any changes to the reference list should be mentioned in the rebuttal letter that accompanies your revised manuscript.

**R: We have revised the reference list.**

------------------------------------------------------------

Requests from Editors:

3. Please remove the information on data availability from the end of the main text: this should be detailed in the submission form (to appear in the article metadata in the event of publication). We were unable to open the specified file, and it may be that some additional information is needed.

**R: We have removed the section and included in the submission form.**

4. In line 2 of the abstract, please adapt the text to "... have been shown to reduce alcohol use" or similar.

**R: We have adapted the text as suggested.**

5. In the abstract, please adapt the sentence summarizing study numbers to "These RCTs evaluated ...".

   **R: We have adapted the text as suggested.**
6. We feel that a few words need to be added to the abstract to explain the nature of "brief interventions". One way to achieve this would be to add a new sentence immediately after that beginning "These RCTs evaluated ..." in which you give 1-2 examples of each category of intervention (e.g., "Brief interventions included ...").

**R: We added a brief definition along with the description of the number of studies per category.**

7. Please adapt the text "Due to high heterogeneity of intervention types ..." in the abstract to state that a meta-analysis was not carried out for this reason (removing that point from the final sentence of the "Methods and findings" subsection).

**R: We have adapted the text as suggested.**

8. In the first point of the Author summary, please make that "high rates" or similar (to avoid the implied comparison of "higher").

**R: We have adapted the text as suggested.**

9. In the second point, please adapt the wording of the second point to "In order to investigate the potential for a patient-level intervention ..." or similar.

**R: We have adapted the text as suggested.**

10. At the end of the Introduction, please move the PICOS sentence to early in the Methods section.

**R: We have adapted the text as suggested.**

11. In the methods section, under "Study selection", it may be helpful to add a few words to clarify the meaning of "six pairs of reviewers" (followed by 11 sets of initials), e.g., "from" the specified individuals; similarly, the "third reviewer" might be "DG or CS".

**R: We have adapted the text as suggested.**

12. In the results section, under "Brief interventions", please avoid "wasn't".

**R: We have adapted the text as suggested and checked for other possible uses of the work "wasn't"**

13. In the results section, under "Biomedical treatments", please correct the apparently broken reference (Correa, 2013).

**R: We have corrected the broken reference**

14. Noting reference 12, please remove the information on version from the citation.

**R: We have corrected the reference**

15. Noting reference 15, please remove all iterations of "[Internet]" from the reference list.

**R: We have corrected the reference**

16. In reference 29, please reverse the current order of study group name and title.

**R: We have corrected the reference**

17. We suggest adapting the title of table 1 to "All randomized controlled studies (75)".

**R: We have adapted the title.**

18. Please convert figure 2 to a supplementary figure.

**R: We have converted as requested**

19. Please confirm that the map in figure 2 can be published under a CC-BY licence (in principle permitting commercial re-use).

**R: Yes, we have confirmed it can be published. It was created using Open access technology.**

Thank you for the opportunity to revise our manuscript.

Catherine A. Staton, MD MScGH

Associate Professor of Emergency Medicine and Global Health

Director, Global Emergency Medicine Innovation and Implementation (GEMINI) Research Lab

EM Director, Hubert Yeargan Global Health Residency and Fellowship Pathway

Division of Emergency Medicine, Department of Surgery

Duke University School of Medicine

Duke Global Health Institute
